# Syndecan-4 Modulates Cell Polarity and Migration by Influencing Centrosome Positioning and Intracellular Calcium Distribution

**DOI:** 10.3389/fcell.2020.575227

**Published:** 2020-10-15

**Authors:** Daniel Becsky, Kitti Szabo, Szuzina Gyulai-Nagy, Tamas Gajdos, Zsuzsa Bartos, Arpad Balind, Laszlo Dux, Peter Horvath, Miklos Erdelyi, Laszlo Homolya, Aniko Keller-Pinter

**Affiliations:** ^1^Department of Biochemistry, Faculty of Medicine, University of Szeged, Szeged, Hungary; ^2^Department of Optics and Quantum Electronics, Faculty of Science and Informatics, University of Szeged, Szeged, Hungary; ^3^Institute of Enzymology, Research Centre for Natural Sciences, Hungarian Academy of Sciences Center of Excellence, Budapest, Hungary; ^4^Institute of Biochemistry, Biological Research Centre, Hungarian Academy of Sciences, Szeged, Hungary

**Keywords:** syndecan-4, proteoglycan, cell polarity, super-resolution microscopy, actin, calcium, centrosome, cell migration

## Abstract

Efficient cell migration requires cellular polarization, which is characterized by the formation of leading and trailing edges, appropriate positioning of the nucleus and reorientation of the Golgi apparatus and centrosomes toward the leading edge. Migration also requires the development of an asymmetrical front-to-rear calcium (Ca^2+^) gradient to regulate focal adhesion assembly and actomyosin contractility. Here we demonstrate that silencing of syndecan-4, a transmembrane heparan sulfate proteoglycan, interferes with the correct polarization of migrating mammalian myoblasts (i.e., activated satellite stem cells). In particular, syndecan-4 knockdown completely abolished the intracellular Ca^2+^ gradient, abrogated centrosome reorientation and thus decreased cell motility, demonstrating the role of syndecan-4 in cell polarity. Additionally, syndecan-4 exhibited a polarized distribution during migration. Syndecan-4 knockdown cells exhibited decreases in the total movement distance during directional migration, maximum and vectorial distances from the starting point, as well as average and maximum cell speeds. Super-resolution direct stochastic optical reconstruction microscopy images of syndecan-4 knockdown cells revealed nanoscale changes in the actin cytoskeletal architecture, such as decreases in the numbers of branches and individual branch lengths in the lamellipodia of the migrating cells. Given the crucial importance of myoblast migration during embryonic development and postnatal muscle regeneration, we conclude that our results could facilitate an understanding of these processes and the general role of syndecan-4 during cell migration.

## Introduction

Cell migration is a fundamentally important factor in various physiological and pathological processes, including morphogenesis, immune surveillance, tissue regeneration, and cancer cell metastasis (Ridley et al., 2003). Cell motility and directed migration require the establishment of cell polarization, defined as the formation of distinct front and rear cellular areas. This process is characterized by the emergence of an actin-mediated lamellipodial membrane protrusion, which forms the leading edge, as well as the development of a retracting tail. The leading edge protrusions depend on polarized intracellular signaling processes. Polarization is also defined by the positioning of the cell nucleus and reorientation of the Golgi network and microtubule organizing center toward the leading edge (Vicente-Manzanares et al., 2005; Zhang and Wang, 2017). Cell motility is orchestrated by the formation of integrin-dependent adhesions to the surrounding matrix and the detachment of these adhesions from distinct regions at the rear of the cell (Lauffenburger and Horwitz, 1996; Ridley et al., 2003). These mechanisms direct the cell motility cycle and are required for cell migration in response to various factors. However, the mechanism by which this motility system integrates extracellular signals with cell polarity and cytoskeletal remodeling to promote directionally persistent migration remains unclear.

Calcium (Ca^2+^) has been identified as an essential factor in cell migration. Ca^2+^ forms an increasing front–rear gradient that is involved in the disassembly of focal adhesions and, consequently, the rear-end retraction and the movement of the cell. This essential front–rear polarity is maintained by restricting the spontaneous formation of lamellipodia at the trailing edges of migrating cells (Tsai et al., 2015; Kim et al., 2016). The steering of membrane protrusions is directed by a localized Ca^2+^ influx created by stretch-activated Ca^2+^ channels in the front of a migrating cell, whereas other types of Ca^2+^ influx have been reported to mediate the detachment of rear protrusions (Kim et al., 2016). However, previous reports describing the coordination of cell migration by the Ca^2+^ gradient have provided limited insights into cell motility and the formation of these gradients.

Syndecans are a family of four transmembrane proteoglycans, each of which comprises a variable N-terminal ectodomain, a highly conserved short transmembrane and a C-terminal cytoplasmic domains (Zimmermann and David, 1999). Three syndecans are distributed in a tissue-specific manner (Xian et al., 2010; Elfenbein and Simons, 2013): syndecan-1 is mainly present in epithelial cells, syndecan-2 is expressed in mesenchymal cell types and developing neural tissues, whereas syndecan-3 is present in neural tissues and the developing musculoskeletal system. In contrast, syndecan-4 is expressed ubiquitously (Xian et al., 2010). Usually, the ectodomains of syndecans contain three heparan sulfate chains attached to a serine residue via tetrasaccharide linkers (Carey, 1997), although syndecan-1 and syndecan-3 possess additional chondroitin sulfate chains. The interactions of the ectodomain with extracellular matrix molecules, fibronectin, matrix metalloproteinases, growth factors and other cell surface receptors (e.g., integrins) activate downstream signaling pathways. The cytoplasmic domain comprises a variable region unique to each member of the syndecan family, as well as two conserved regions that interact with four-point-one, ezrin, radixin, and moesin (FERM) proteins; Src kinase; and cortactin (Granes et al., 2003). In syndecan-4, the variable region binds and activates the catalytic domain of protein kinase C α (PKCα) (Koo et al., 2006), as well as directly binds α-actinin in a beta-integrin-independent manner (Greene et al., 2003). The ability of syndecan-4 to link the extracellular matrix and cytoskeleton enables this proteoglycan to contribute to several outside-in and inside-out signaling events, such as the sequestration and concentration of matrix components, as well as effects on cell–matrix adhesion, endocytosis, exosome biogenesis or cytokinesis (Keller-Pinter et al., 2010; Elfenbein and Simons, 2013; Afratis et al., 2017). Syndecan-4 also regulates the activity of the small GTPase Rac1 (Bass et al., 2007; Keller-Pinter et al., 2017) and the level of intracellular Ca^2+^ (Liu et al., 2012; Gopal et al., 2015), and contributes to the phosphorylation of focal adhesion kinase (FAK) (Wilcox-Adelman et al., 2002).

Syndecans play an important role in tissue regeneration (Chung et al., 2016). For example, the skeletal muscle is renewed constantly in response to injury, exercise or muscle diseases. During the repair process, activated stem (i.e., satellite) cells form myoblasts that proliferate, migrate to the injured site, differentiate and fuse into polynuclear myotubes (Schultz and McCormick, 1994; Hawke and Garry, 2001). Syndecan-4 is a cell surface marker of both quiescent and proliferating satellite cells (Cornelison et al., 2001). Although syndecan-4 knockout mice cannot regenerate damaged muscle tissue (Cornelison et al., 2004), the details of the underlying mechanism remain unknown. Previously, we reported that syndecan-4 affects myoblast proliferation by modulating myostatin signaling and the G1/S transition in cell cycle (Keller-Pinter et al., 2018), and directional persistence of random cell migration is affected by syndecan-4-mediated Tiam-1 expression and distribution (Becsky et al., 2020). In this study, we demonstrated that syndecan-4 knockdown induced nanoscale alterations in the lamellipodial actin fiber structure of migrating myoblasts. Moreover, we found that syndecan-4 distributes asymmetrically during cell migration and determines cellular polarity by influencing the positioning of centrosomes and the development of the front–rear Ca^2+^ gradient. Although several previous reports have described a role for syndecan-4 in cell migration, here we present a super-resolution structure of the actin cytoskeleton. Moreover, this is the first report to describe the role of syndecan-4 in the development of the Ca^2+^ gradient and centrosome positioning in a migrating cell.

## Materials and Methods

### Cell Culture and Plasmids

C2C12 mouse myoblast cells (ATCC; Manassas, VA, United States) were cultured in high-glucose Dulbecco’s modified Eagle’s medium containing 4.5 g/L glucose, 584 mg/L glutamine and 110 mg/L pyruvate (Corning, NY, United States) supplemented with 65 μg/mL gentamicin (Lonza, Basel, Switzerland), and 20% fetal bovine serum (Gibco/Thermo Fisher Scientific, Waltham, MA, United States). To achieve syndecan-4 knockdown, C2C12 cells were transfected stably with plasmids expressing short hairpin RNAs (shRNAs) specific for mouse syndecan-4 (shSDC4#1 and shSDC4#2) or a scrambled target sequence. The plasmids were obtained from OriGene (TR513122; Rockville, MD, United States) and targeted the following sequences: 5’-GAA CTG GAA GAG AAT GAG GTC ATT CCT AA-3’ (shSDC4#1), 5’-GCG GCG TGG TAG GCA TCC TCT TTG CCG TT-3’ (shSDC4#2) and 5’-GCA CTA CCA GAG CTA ACT CAG ATA GTA CT-3’ (scrambled). X-tremeGENE transfection reagent (Roche, Basel, Switzerland) was used for the transfection procedures. Transfected cells were then selected in medium containing 4 μg/mL puromycin (Sigma-Aldrich, St. Louis, MO, United States).

### Time-Lapse Imaging of Live Cells

Cells were seeded into the reservoirs of 2-well cell culture silicon inserts at a density of 3 × 10^4^ cells/well (Ibidi, Martinsried, Germany). The inserts were designed to ensure directional cell migration, with a defined cell-free gap of 500 μm. Upon cellular attachment, the medium was replaced with serum-reduced medium for 24 h to suppress cell division. After nuclear staining with Hoechst 33342 (0.5 μg/mL) for 1 h and washing with PBS, the insert was removed and the migration of cells into the cell-free zone was screened. Time-lapse images were captured in 20 min intervals for 8 h at 37°C and 5% CO_2_ using the PerkinElmer Operetta (PerkinElmer, Inc., Waltham, MA, United States) high-content imaging system with a 20 × objective (20 × long WD; NA = 0.45, working distance: 7.8 mm; field of view: 675 × 509; depth of focus: 4.6 μm; optical xy resolution: 0.7 μm).

### Single-Cell Tracking of Cultured Myoblasts

Time-lapse microscopy was used to quantify the migratory parameters. Single cells were tracked manually from frame to frame using the ImageJ (National Institutes of Health, Bethesda, MD, United States)^1^ and CellTracker^2^ (Piccinini et al., 2016) software programs. Nuclear tracking was used to follow the migration of individual cells. Dying or damaged cells were excluded from the analysis. The length of total path, maximum distance from the origin, as well as the average and maximum cell speeds were calculated. The vectorial distance of migration (i.e., real shift of the cell) from the origin was also quantified. Individual migratory tracks into the cell-free zone were visualized.

### Wound Scratch Assay

For the wound scratch assay, cells were grown in 6-well plates until they reached confluence. After 24 h incubation in serum-reduced medium, cell-free zones were created by scratching the cell layer with a P200 pipette tip. Images of the cell-free zone were captured immediately (0 h), 4 and 8 h after wounding, using a Leica DMi1 phase-contrast microscope (Leica Microsystems, Wetzlar, Germany). Between imaging periods, the cells were incubated at 37°C and 5% CO_2_. The area of the cell-free zone was measured using Digimizer image analysis software (MedCalc Software bvba, Ostend, Belgium). The closure of the cell-free area was calculated as follows: (area of cell-free zone at t_0h_ - area of cell-free zone at t_*xh*_)/area of cell-free zone at t_0h_.

### Fluorescence Staining

For fluorescence cytochemistry, the cells subjected to wounding were fixed at indicated time points, stained with fluorescence markers, and studied to evaluate the migratory cells in the scratched area. For centrosome staining, cells were fixed with methanol 2, 4, and 6 h after scratching. After permeabilization with 0.5% Tween-20 (Sigma-Aldrich), the samples were blocked in 4% bovine serum albumin (BSA; Sigma-Aldrich), and stained with a mouse monoclonal anti-γ-tubulin antibody (1:200; Sigma-Aldrich) at 4°C overnight, followed by incubation with an Alexa Fluor 488-conjugated anti-mouse secondary antibody (Jackson ImmunoResearch, Cambridgeshire, United Kingdom) a day later.

To visualize the actin filaments, cells subjected to the above-described scratch assay were fixed with a methanol-free 4% formaldehyde solution (Thermo Fischer Scientific) 2 h after wounding. After permeabilization with 0.3% Triton X-100 (Sigma-Aldrich) and blocking in 4% BSA (Sigma-Aldrich), the actin filaments were stained with Alexa Fluor 647-conjugated phalloidin (A22287, Thermo Fisher Scientific).

For syndecan-4 immunostaining, myoblasts were fixed with 4% formaldehyde solution 2 h after wounding, permeabilized with 0.3% Triton X-100, and blocked with 1% BSA. Rabbit polyclonal anti-syndecan-4 primary antibody (immunogen: synthetic peptide surrounding amino acid 184 of human syndecan 4; PA1-32485; Invitrogen, Carlsbad, CA, United States) was visualized with the appropriate Alexa Fluor 568-conjugated (Invitrogen), or Alexa Fluor 488-conjugated secondary antibody (Jackson ImmunoResearch, Cambridgeshire, United Kingdom) secondary antibody. For double immunostaining experiments, cells were fixed with 4% formaldehyde solution, permeabilized with 0.1% Triton X-100 and blocked with 3% BSA. Focal adhesions were marked with mouse monoclonal anti-FAK primary antibody (sc-271126; Santa Cruz Biotechnology, Dallas, TX, United States) and with Alexa Fluor 488-conjugated secondary antibody (Jackson ImmunoResearch, Cambridgeshire, United Kingdom). The *cis-*Golgi network was stained by mouse monoclonal anti-GM130 antibody (610822; BD Biosciences, San Jose, CA, United States), and followed by incubation with CF568-conjugated secondary antibody (Biotinum, Fremont, CA, United States). Nuclei were counterstained with Hoechst 33258 (0.01 mg/mL, Sigma-Aldrich).

### Quantification of Centrosome Positioning

The positions of centrosomes were analyzed to quantify cell polarity, based on a previous characterization of centrosome reorientation in response to a scratch (Etienne-Manneville and Hall, 2001). Anti-γ-tubulin-stained samples were inspected and imaged using a Nikon Eclipse Ti-E microscope frame (Nikon Instruments Inc., Melville, NY, United States) with epifluorescent illumination using 20 × objective (Nikon Plan fluor 20 × DIC N2, NA = 0.50). The images were analyzed using ImageJ software.

Two hours after wounding, only the migrating cells next to the scratched area were analyzed. For selected cells adjacent to the cell-free zone, the direction of migration was designated as perpendicular to the wound edge, the nucleus was set as the origin, and a 30°circular sector facing the direction of wound closure was assigned. Centrosomes situated within this assigned area were scored as correctly oriented. To monitor the time dependency of centrosome reorientation in different cell lines, the position of centrosomes was analyzed 2, 4, and 6 h after wounding in the 1st and 2nd row of myoblasts in the different cell lines along the wound edge based on the method described by Gotlieb et al. (1983). The position of centrosomes was considered “toward” the wound edge (between the nucleus and the wound edge), “middle” (along the side the nucleus), or “away” (between the nucleus the monolayer behind the cells).

### Super-Resolution dSTORM Imaging

Super-resolution direct stochastic optical reconstruction microscopy (dSTORM) measurements were performed using a custom-made inverted microscope based on a Nikon Eclipse Ti-E frame. After conditioning (through spatial filtering via fiber coupling and beam expansion), the applied laser beams were focused into the back focal plane of the microscope objective (Nikon CFI Apo 100 ×, NA = 1.49) to produce a collimated beam on the sample. The angle of illumination was then set through a tilting mirror mounted into a motorized gimbal holder and placed into the conjugate plane of the sample. All dSTORM images were captured under epi-illumination at an excitation wavelength of 634 nm (Thorlabs HL63133DG: 637 nm, P_max_ = 170 mW in a Thorlabs TCLDM9 TE-Cooled mount set to 19°C). The laser intensity was controlled via a Thorlabs LDC500 laser driver and set to an output of 2–4 kW/cm^2^ on the sample plane. An additional laser (Nichia: 405 nm, P_max_ = 60 mW) was used for reactivation. Images were captured using an Andor iXon3 897 BV EMCCD digital camera (512 pixels × 512 pixels; pixel size: 16 μm). The size of the illuminated sample region was matched to the size of the detector, which determined the field of view (80 × 80 μm^2^). Typically, the frame stacks for dSTORM super-resolution images were captured at a reduced image size (i.e., crop mode). A fluorescence filter set (Semrock, LF405/488/561/635-A-000 dichroic mirror with a BLP01-647R-25 emission filter) was used to select and separate the excitation and emission lights in the microscope. During measurements, the perfect focus system of the microscope was used to maintain focus on the sample at a precision level of < 30 nm. Immediately before measurement, the sample storage buffer was replaced with a GLOX switching buffer (van de Linde et al., 2011), and the sample was mounted on a microscope slide. During a typical imaging session, 20,000 frames were captured at an exposure time of 20 or 30 ms. The image stacks were analyzed using rainSTORM localization software (Rees et al., 2013) and reconstructed using the built-in Simple Histogram method with a super-pixel size of 13.33 nm. The Thompson-precision (Thompson et al., 2002) and PSF size acceptance ranges were set to 0–35 nm and 0.7–1.5 pixels, respectively.

### Nanoscale Analysis of the Actin Cytoskeletal Structure

After dSTORM imaging, phalloidin-stained samples were subjected to a nanoscale analysis of the actin cytoskeleton. The dSTORM images of lamellipodial actin structures were processed using ImageJ software. The super-resolution images were converted to grayscale, adjusted to a fixed threshold, and noise filtered. The ImageJ Skeletonize function was used to create binary skeletonized images. Then the Skeleton Analysis plugin was used to calculate the number of branches belonging to each skeleton in every image and to measure the length of each individual branch. To describe the difference between the cortical actin-rich region and the inner actin-depleted area of the lamellipodial actin network, three areas (each 126 × 124 px) were randomly selected in the external region (with a width of 350 px beneath the plasma membrane) and three in the inner, internal region of the lamellipodia. Then the average number of branches and average length of the individual branches were measured in each of these selected rectangles and compared.

### Evaluation of Syndecan-4 Immunostaining

Wide-field fluorescence images of syndecan-4 immunostained samples were acquired by a Nikon Eclipse Ti-E microscope (Nikon Instruments Inc.) with 40 × (Nikon CFI Plan Fluor 40 ×, NA = 0.75) and 100 × (Nikon CFI Plan Apo DM Lambda 100 × Oil, NA = 1.45) objectives, and pseudo-colored using ImageJ. The contours of the individual cells were drawn, and the average pixel intensity within the border of the cells were quantified following background correction. The intensity value of each pixel was measured within the selected area and the sum of the intensities was divided by the area of the cell to obtain the average syndecan-4 intensity value of the individual cells. Furthermore, cells were partitioned into 4 quadrants considering the nucleus as the origin, a 90°circular sector facing the direction of the wound closure was assigned, and the syndecan-4 signal intensity within this area was quantified.

### Assessment of Intracellular Ca^2+^ Distribution

As control, scrambled and two syndecan-4-targeted myoblast cell lines were seeded onto glass 8-well chambered coverslips (ibidi GmbH, Gräfelfing, Germany) at 1 × 10^4^ cells/well density and grown for 24 h in serum-reduced medium. The confluent cultures were scratched as described above and further incubated for 2 h. Subsequently, the cells were subjected to 2 μM Fluo-4 AM and 3 μM Fura Red AM (Thermo Fisher Scientific) in serum-free D-MEM containing 50 μM Verapamil (Sigma) for 30 min at 37°C and 5% CO_2_. Verapamil was included to block the activity of multidrug transporters hindering effective dye loading. After several thorough washing steps, the green (493–572 nm) and far red (609–797 nm) fluorescence images were simultaneously acquired at 488 and 458 nm excitations, respectively, using a Zeiss 710 LSM laser scanning fluorescence confocal microscope with a Plan-Apochromat 40 × (N.A. = 1.4) oil immersion objective. The images were analyzed by ImageJ 1.49g software (National Institutes of Health, Bethesda, MD, United States). Ratio images were generated using the Ratio Plus Plug-in. For quantitative analysis, the Fluo-4 and Fura Red fluorescence intensities were determined along the axis of migrating cells starting from the leading edge. After background correction, ratios of green and red fluorescence were calculated. The slope of the intracellular Ca^2+^ distribution was determined by least squares method.

### Statistical Analysis

Differences between groups were analyzed using a one-way ANOVA, followed by the Scheffe *post hoc* test or Student’s *t*-test. GraphPad Prism 7.0 (GraphPad Software Inc., San Diego, CA, United States) was used for graphing and statistical analyses. The data are expressed as means + standard errors of the means. A *p* < 0.05 was considered significantly different.

## Results

### Syndecan-4 Knockdown Decreases Directional Cell Migration

Initially, we evaluated the expression of syndecan-4 in C2C12 myoblasts transfected stably with plasmids expressing shRNA specific for syndecan-4 (shSDC4#1 and SDC4#2 cell lines) using Western blotting technique. A more significant reduction in syndecan-4 expression was observed in shSDC4#1 cells vs. shSDC4#2 cells, whereas the scrambled sequence had no effect on syndecan-4 level (Supplementary Figure 1).

**FIGURE 1:**
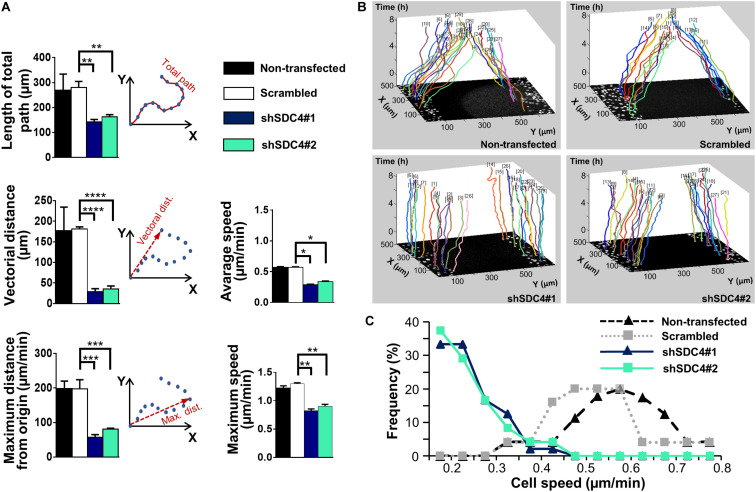
The role of syndecan-4 in the directional migration of myoblasts. **(A)** Migration of non-transfected, scrambled, and syndecan-4-silenced (shSDC4#1 and shSDC4#2) C2C12 myoblasts to a cell-free zone was assessed after the removal of a cell culture insert. The total length of movement, maximum distance from the starting point, vectorial distance (i.e., real displacement of the cells), and the average and maximum cell speeds during directional migration are presented. The total duration of live cell microscopy was 8 h, at a frame rate of 3/1 h. Four independent experiments were conducted, with 60–87 cells/cell line and 5–6 fields of view/experiment. Data are presented as means + standard errors of the means; *p < 0.05, **p < 0.01, ***p < 0.001, and ****p < 0.0001. **(B)** Representative three-dimensional migration tracks. Different colors represent the total migrations of individual myoblasts; x and y axes: position of the cell (μm), *z*-axis: time (h). **(C)** Histograms depict the distributions of cells from different lines according to cell speed (intervals of 0.05 μm/min). The frequencies of cells from each line with average speeds within each interval were evaluated and are presented on the *y*-axis.

We then measured the effect of syndecan-4 knockdown on directional migration *in vitro* into cell-free zones created using cell culture inserts for an 8 h period (Supplementary Movies 1–4). During this analysis, we observed significant decreases in the length of total movement, the vectorial distance, the maximum distance from the origin, as well as the average and maximum cell speeds in both the shSDC4#1 and shSDC4#2 cell lines (Figure 1A), whereas no significant difference was observed between the non-transfected and scrambled cell lines (Figure 1A). Moreover, we observed a greater reduction in migratory parameters in shSDC4#1 cells (Figure 1), consistent with the previous observation of greater syndecan-4 suppression in this line. An evaluation of the migratory tracks of individual cells depicts the positions of the x and y coordinates corresponding to the paths taken by each cell during the indicated time (as z; Figure 1B). The migratory tracks of highly motile control cells crossed each other in the middle of the cell-free zone (black area in the center of each image), whereas those of syndecan-4 knockdown cells hardly moved from the original x-y positions during the 8 h experimental period. We then prepared histograms to depict the percentages of cells within each velocity range (Figure 1C). Notably, the histograms of the non-transfected and scrambled cells formed bell-shaped curves, whereas those of both silenced cell lines exhibited a left-skewed distribution suggesting the higher ratio of less motile cells.

**FIGURE 2:**
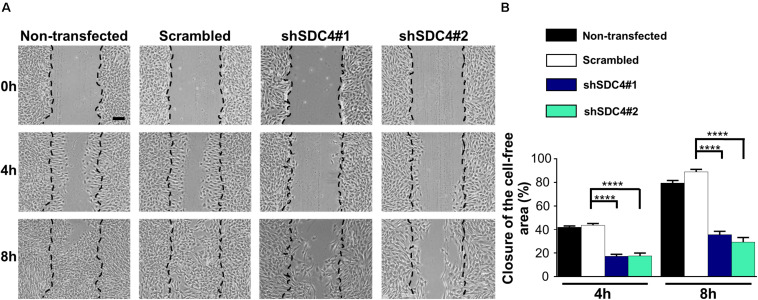
Syndecan-4 influences the closure of the cell-free zone. **(A)** Representative microscopy images taken 0, 4, and 8 h after the initiation of a wound scratch assay. Dashed lines indicate the position of the cell-free zone at 0 h. Scale bar: 200 μm. **(B)** Quantification of the closure of the cell-free area in cultures of non-transfected, scrambled, and syndecan-4-silenced (shSDC4#1 and shSDC4#2) cells; *n* = 4 independent experiments. Data are shown as means + standard errors of the means; ****p < 0.0001.

Representative images in Figure 2A depict a scratch wound in a confluent culture at 0, 4, and 8 h. Quantification of the wound closures revealed a reduced closure of the cell-free zone in both syndecan-4 knockdown lines (Figure 2B). No significant difference was observed between non-transfected and scrambled cells (Figure 2B).

### Syndecan-4 Affects the Nanoscale Architecture of the Actin Cytoskeleton, as Determined by Super-Resolution dSTORM

**FIGURE 3:**
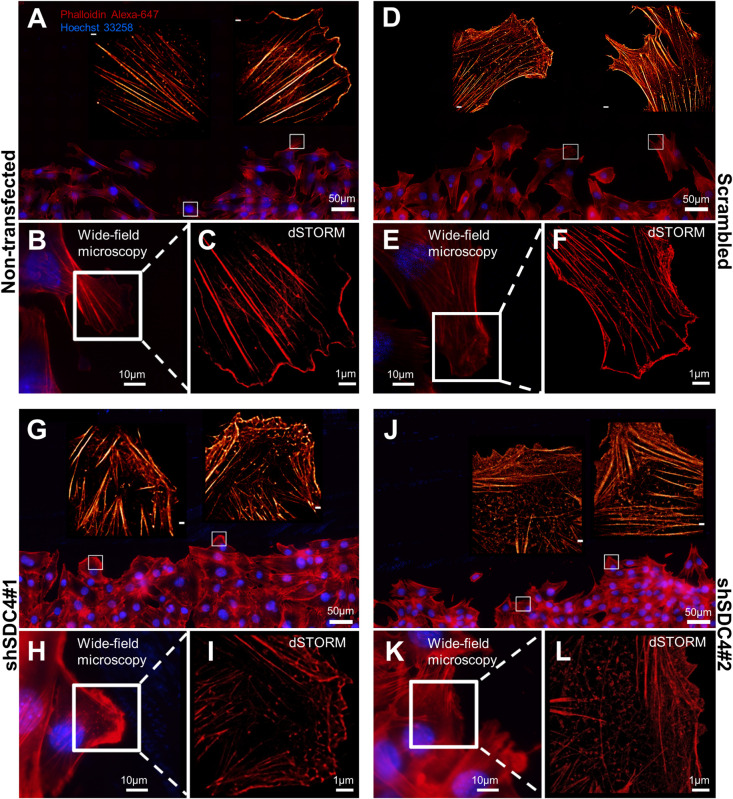
Direct stochastic optical reconstruction microscopy (dSTORM) analysis of the actin cytoskeleton after syndecan-4 silencing. Representative wide-field fluorescence and super-resolution dSTORM images depict the actin skeletons of the cells adjacent to the cell-free zone in cultures of non-transfected **(A–C)**, scrambled **(D–F)**, shSDC4#1 **(G–I),** and shSDC4#2 **(J–L)** cell lines. Confluent monolayers were subjected to wound scratching. The cells were fixed 2 h later, and the actin filaments were stained with Alexa Fluor 647-conjugated phalloidin (red). Wide-field fluorescence images were obtained around the cell-free zone (**A,D,G,J,** higher magnification: **B,E,H,K**). Full panoramic maps of the scratched areas are shown in Supplementary Figures 3–6. The insets of the wide-field fluorescence images depict dSTORM images of the lamellipodial regions of migrating cells adjacent to the cell-free zone **(A,B,D,E,G,H,J,K).** Representative dSTORM images of lamellipodial actin structures are embedded in the original low-magnification images (**A,D,G,J**; bar: 1 μm) or are shown in separate higher magnification panels **(C,F,I,L)**. Nuclei are stained by Hoechst 33258 (blue).

Cell motility is regulated by both extracellular factors and internal signaling mechanisms, including actin cytoskeletal remodeling. As syndecan-4 plays a crucial role in the organization of the actin cytoskeleton (Baciu et al., 2000; Elfenbein and Simons, 2013; Cavalheiro et al., 2017), we evaluated actin filaments using wide-field fluorescence microscopy (Figures 3A,B,D,E,G,H,J,K) and single-molecule localization super-resolution dSTORM imaging (lower magnification: Figures 3A,D,G,J; higher magnification: Figures 3C,F,I,L). Notably, our super-resolution dSTORM images reveal the sub-diffraction structure of the actin cytoskeleton and enable a more sophisticated experimental comparison of control and syndecan-4 knockdown samples. The reduced fluorescence background and enhanced resolution enabled visualization of the orientations and densities of individual actin bundles.

**FIGURE 4:**
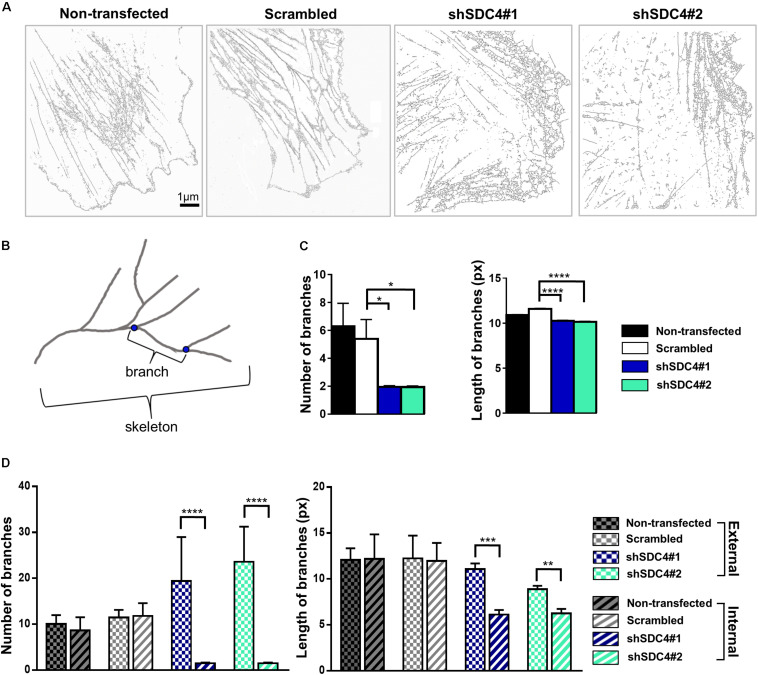
Skeletal analysis of dSTORM images of the lamellipodial actin network. The phalloidin-stained lamellipodial actin cytoskeletons of non-transfected, scrambled, and syndecan-4-silenced (shSDC4#1 and shSDC4#2) cells were analyzed. Representative binary images converted from dSTORM images of the actin cytoskeleton are shown **(A)**. Within the actin network, branching points divide the skeletons into smaller branches **(B)**. The number of branches in the skeletons and the lengths of individual branches in the lamellipodial actin network in the whole binary images were quantified **(C)**. To measure the differences between the external and the internal region of the lamellipodial actin network, the average number and length of branches were compared in randomly selected areas (three areas in both external and internal regions, each 126 × 124 pixels in size) of the binary images **(D)**. Binary images of 5 cells/cell line were studied. Numbers of analyzed skeletons: 5,560–8,450/cell line; numbers of analyzed branches: 26,723–32,813/cell line. Number of analyzed skeletons in a single 126 × 124 pixels area: 2–69, number of analyzed branches in a single 126 × 124 pixels area: 32–336. Data are shown as means + standard errors of the means; *p < 0.05, **p < 0.01, ***p < 0.001, and ****p < 0.0001.

Next, wound scratch assays were performed to study the lamellipodial actin networks in migrating cells. To prove the migratory phenotype of the cells next to the cell-free zone, we stained the focal adhesions by anti-FAK antibody in the different cell lines, and FAK-stained focal adhesions were observed at the end of the stress fibers (Supplementary Figure 2). Interestingly, both the size and the number of focal adhesions decreased in syndecan-4 knockdown cells (Supplementary Figure 2). The cells next to the scratched areas were analyzed after actin filament labeling of the samples. For every sample, a panoramic map of individual wide-field fluorescence images was generated to cover the whole area of cell culture around the scratch wound (Supplementary Figures 3–6), and the lamellipodia of the migrating cells next to the wound were analyzed by dSTORM. Representative areas of the panoramic maps are shown in Figures 3A,D,G,J. Notably, syndecan-4 silencing altered the organization of the actin cytoskeleton (Figure 3) by hindering the development of actin structures (Figures 3G–L). The non-transfected and scrambled cells exhibited well-developed actin filaments (Figures 3A–F), whereas this filamentous actin cytoskeletal structure was less pronounced, and the lamellipodial actin network was less organized in syndecan-4 knockdown cells (Figures 3G–L). Next, dSTORM images of the actin cytoskeleton were converted to binary images (Figure 4A) and analyzed further to quantify nanoscale changes in the actin network (Figure 4B). An analysis of binary images of the lamellipodial actin filaments (Figure 4A) revealed decreases in both the number of branches and the lengths of individual branches in the lamellipodial actin networks of syndecan-4 knockdown cells (Figure 4C).

As the binary images suggested the presence of an actin-depleted inner region some micrometers away from the leading edge in syndecan-4 knockdown cell lines, next we quantified the nanoscale changes of the cortical (external) and the inner area of the lamellipodial actin network in the cell lines (Figure 4D). Both the average number of branches (in each skeleton) and the lengths of individual branches decreased in the inner region as compared to the external region of the lamellipodia in syndecan-4 knock-down cells, indicating the inhomogeneous lamellipodial actin structure in these cells (i.e., actin-rich external region and actin-depleted inner area).

### Syndecan-4 Affects Centrosome Positioning and Cell Polarity

**FIGURE 5:**
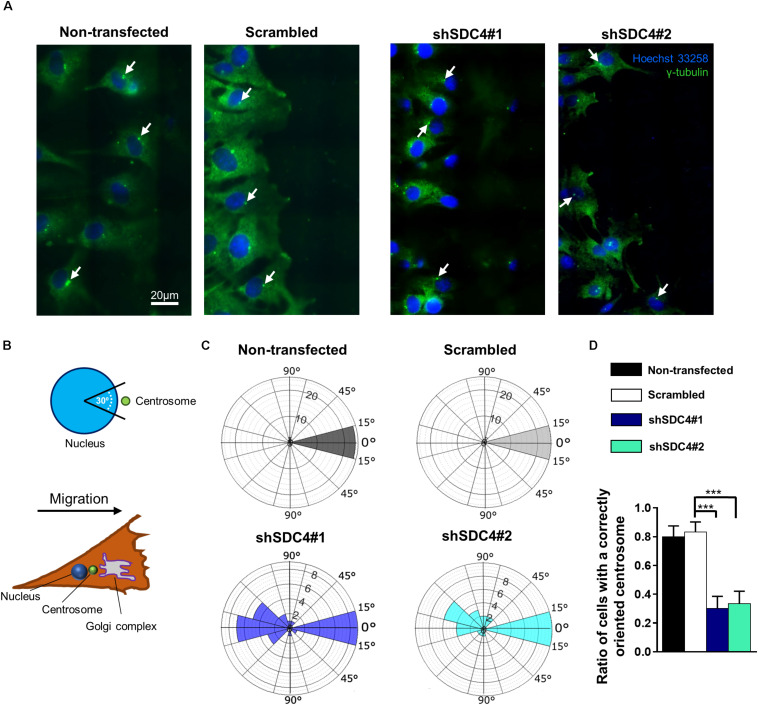
Syndecan-4 affects centrosome positioning during migration. **(A)** Representative wide-field fluorescence images of the studied cell lines depict the positions of centrosomes 2 h after scratching. Anti-γ-tubulin-labeled centrosomes and Hoechst 33258-stained nuclei are shown in green and blue, respectively. Arrows indicate the centrosomes. **(B)** Schematic representation of a polarized migratory cell. To quantify the positions of centrosomes, the nucleus was set as the origin, and centrosomes located in the 30°circular sector facing toward the direction of wound closure were considered properly located. **(C)** Pie charts (i.e., polar histograms) show the localization of centrosomes in different cell lines. The plane was partitioned into 30°circular sectors with the nucleus as the origin. The radius of each circular sector represents the number of cells with centrosomes located in that 30° sector. *N* = 3 independent experiments. Thirty cells were analyzed per cell line. **(D)** Quantification of the results shown in **(C)**. The graph presents the ratios of centrosomes in the 30° sector facing the cell-free area. Data are shown as means + standard errors of the means; ***p < 0.001.

Appropriate polarization of the cell (Lauffenburger and Horwitz, 1996), adequate positioning of the cellular compartments (Petrie et al., 2009), and dynamic reconstruction of the actin cytoskeleton (Gardel et al., 2010; Parsons et al., 2010) are required for efficient cell migration. As syndecan-4 silencing was shown to reduce myoblast migration, we next studied the polarization of syndecan-4 knockdown cells using centrosome localization, an indicator of cell polarity in migrating cells (Etienne-Manneville and Hall, 2001; Zhang and Wang, 2017). Specifically, the exact positions of the centrosomes were observed on immunostained samples obtained 2, 4, and 6 h after a wound scratch assay (Figure 5A, Table 1, and Supplementary Figures 7–9). Fluorescence images were captured after centrosome (anti-γ-tubulin) staining and used to generate panoramic maps of the entire scratched area (Supplementary Figures 7–9).

**TABLE 1:** Comparison of the effect of syndecan-4 silencing on centrosome reorientation in the 1st and 2nd row of myoblasts along the wound edge.

	Time after scratch											
	2 h				4 h				6 h			
	Non-transfected	Scrambled	shSDC4#1	shSDC4#2	Non-transfected	Scrambled	shSDC4#1	shSDC4#2	Non-transfected	Scrambled	shSDC4#1	shSDC4#2
**Toward**												
1st	80 ± 4.0	83 ± 1.0	8 ± 1.0	7 ± 1.5	86 ± 2.5	88 ± 1.0	15 ± 1.0	14 ± 2.0	94 ± 4.0	92 ± 3.0	27 ± 3.5	25 ± 3.5
2nd	84 ± 1.5	87 ± 2.5	6 ± 1.5	4 ± 0.5	84 ± 0.5	89 ± 5.0	12 ± 1.0	16 ± 2.0	97 ± 2.0	98 ± 1.5	35 ± 2.5	27 ± 2.5
**Middle**												
1st	8 ± 1.0	8 ± 1.5	22 ± 2.0	20 ± 3.0	2 ± 4.5	4 ± 2.0	17 ± 2.5	19 ± 3.0	2 ± 2.0	5 ± 1.5	16 ± 1.5	23 ± 1.0
2nd	6 ± 0.5	7 ± 1.0	18 ± 3.0	15 ± 5.5	7 ± 6.5	5 ± 8.0	21 ± 1.0	14 ± 3.0	2 ± 2.5	1 ± 0.5	17 ± 2.5	12 ± 1.5
**Away**												
1st	12 ± 3.0	9 ± 2.5	70 ± 3.0	73 ± 4.5	12 ± 2.0	8 ± 1.0	71 ± 1.5	67 ± 1.0	4 ± 2.0	3 ± 1.5	57 ± 2.0	52 ± 2.5
2nd	10 ± 1.0	6 ± 1.5	76 ± 1.5	81 ± 5.0	9 ± 6.0	11 ± 3.0	67 ± 2.0	70 ± 1.0	1 ± 0.5	1 ± 1.0	52 ± 5.0	61 ± 1.0

*Four independent experiments, n = 100–100 cells/cell lines in each row.*

Two h after wounding, cells adjacent to the cell-free area were investigated using the nuclei as the points of reference (i.e., origins). The areas around the nuclei were divided into 30° sectors, and centrosomes located in the 30°circular sector facing toward the cell-free area were considered properly oriented (Figure 5B). Figure 5C depicts the numbers of centrosomes in the various sectors from experiments involving the different cell lines. Notably, syndecan-4 knockdown was associated with significantly fewer centrosomes in the 30°circular sector facing toward the cell-free zone, indicating an improper reorientation of the centrosomes in these cells (Figures 5C,D). In contrast, nearly all centrosomes of the scrambled and non-transfected cells were localized to this 30°circular sector facing toward the cell-free area, indicating precise and proper regulation of centrosome positioning in these controls (Figures 5C,D). There was no significant difference between the non-transfected and scrambled cells (Figure 5D). To analyze the time dependency of centrosome reorientation, the position of centrosomes was studied 2, 4, and 6 h after wounding (Table 1). The number of centrosomes facing the wound edge increased in all cell lines during the 6 h period in both 1st and 2nd row. Analysis of centrosome position along the wound edge revealed that in 83% of the scrambled cells in the first row the centrosomes were located toward the wound edge (between the nucleus and the wound edge) 2 h after wounding and 94% of the cells 6 h following wounding (Table 1). In contrast, only 25–27% of the syndecan-4 silenced cells presented centrosomes with “toward” position 6 h after wounding. In scrambled cells, only a few number of cells exhibited “middle” (along the side the nucleus), or “away” (between the nucleus the monolayer behind the cells) localized centrosomes 6 h after scratching. Based on these results, the reorientation of centrosomes during migration is delayed in syndecan-4 knockdown cells.

### Polarized Distribution of Syndecan-4 During Migration

**FIGURE 6:**
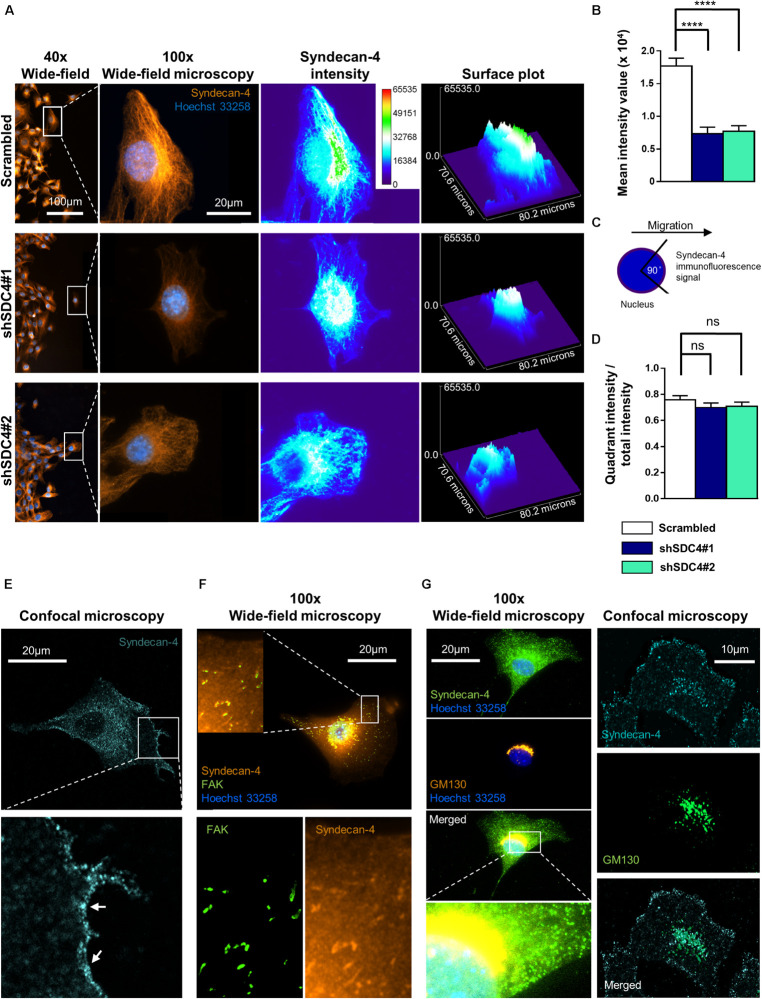
Asymmetric distribution of syndecan-4 in migrating myoblasts. **(A)** Representative images show syndecan-4 distribution following staining with Alexa Fluor 568 fluorophore (orange). Nuclei are stained by Hoechst 33258 (blue). Representative pseudo-color images (2D and 3D) depict syndecan-4 signal intensity as indicated by the calibration bar. **(B)** The mean intensity values of the cells were quantified. **(C)** Cells were partitioned into 4 quadrants considering the nucleus as the origin; and a 90°circular sector facing the direction of the wound closure was assigned and the syndecan-4 signal intensity within this area was quantified. **(D)** The ratio of signal intensity of the quadrant pointing into the direction of migration (see schematic figure, **C**), and the total syndecan-4 intensity of the cell was calculated and compared in the different cell lines. Data are reported as means + standard errors of the means, *n* = 30 cells/cell line were analyzed; ns: not significant; ****p < 0.0001. **(E)** Representative confocal image depicts the cell membrane localization (arrows) of syndecan-4 in a migrating scrambled cell. **(F)** Representative wide-field fluorescence image of syndecan-4 and FAK staining in a migrating scrambled cell. **(G)** Representative wide-field fluorescence and confocal image of GM130 (*cis*-Golgi marker) and syndecan-4 double staining in migrating scrambled cells.

The former experiments demonstrated that syndecan-4 influences cellular polarity indicated by the impaired centrosome positioning and migration properties of myoblasts. Next we examined the intracellular distribution of syndecan-4 in control (scrambled) and syndecan-4 silenced cell lines in wide-field fluorescence images. According to immunostaining experiments, the amount of syndecan-4, considering all fluorescence signal intensities, was significantly higher in control cells than in syndecan-4 silenced cell lines (Figures 6A,B). Syndecan-4 accumulates in the quadrant of the migrating cells facing the wounded area (Figure 6A) which points the direction of migration (Figure 6C). Comparing the amount of syndecan-4 accumulated in the quadrant facing the wounded area (Figure 6C) to the total of syndecan-4 level of the cells did not depict significant difference between the cell lines (Figure 6D). Based on these results, the distribution of syndecan-4 does not change as a result of silencing; only the total amount of syndecan-4 is lower in knockdown cells.

Since the wide-field images showed cytoplasmic syndecan-4 staining, next we performed confocal imaging. The representative confocal image (Figure 6E) depicts the weak cell membrane localization of syndecan-4 in a migrating cell. Since earlier we showed the co-localization of syndecan-4 with the anti-GM130 Golgi marker and syndecan-4 is a member of focal adhesions, next we tested the co-distribution of syndecan-4 with FAK and GM130 (Figures 6F,G). The observed localization of syndecan-4 in the focal adhesions and *cis*-Golgi (Figures 6F,G) can explain the vacuolar and punctate signals of syndecan-4 staining. Moreover, earlier we have shown that the phospho-(Ser179 in human, Ser183 in mouse) syndecan-4 accumulates in the cytoplasm during cytokinesis (Keller-Pinter et al., 2010). Therefore, we cannot exclude, that the syndecan-4 signal in our migrating cells partially originates from the cytoplasmic phosphorylated form.

### Syndecan-4 Knockdown Abrogates the Intracellular Ca^2+^ Gradient in Migrating Cells

**FIGURE 7:**
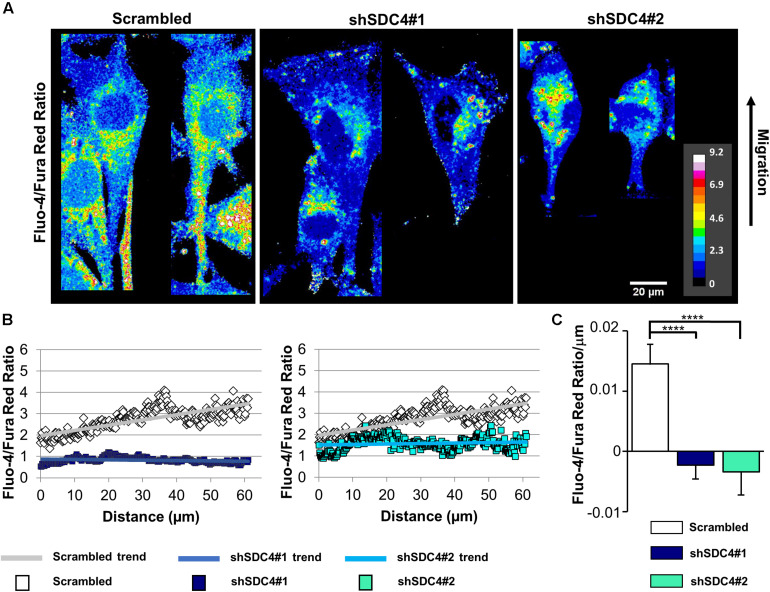
Effect of syndecan-4 silencing on the distribution of intracellular Ca^2+^ in migrating myoblasts. **(A)** The ratio of Fluo-4 and Fura Red fluorescence, an indicator of the intracellular Ca^2+^ level, is shown in the representative pseudo-color images of scrambled, shSDC4#1, and shSDC4#2 cells. **(B)** The ratio of Fluo-4 and Fura Red fluorescence was determined along the migration axis from the leading edge to the rear of cells following scratch wounding. The mean fluorescence ratios are presented as a function of the distance from the leading edge. **(C)** The slopes of Fluo-4/Fura Red ratios along the migration axis in scrambled and syndecan-4 knockdown cells. Migrating cells next to the cell-free zones (*n* = 8–12) revealed that syndecan-4 knockdown completely abolished Ca^2+^ gradient development in migrating cells. Data are shown as the means + standard errors of the means; ****p < 0.0001.

Normally, migrating cells exhibit a gradual increase in Ca^2+^ levels along the axis of migration. Accordingly, we next assessed the distribution of intracellular Ca^2+^ in syndecan-4-silenced C2C12 cells and compered to that seen in cells transfected with a scrambled target sequence. The front–rear Ca^2+^ distribution was studied in cells adjacent to the cell-free area in a scratch-wounded confluent culture (Figure 7A). As expected, the intracellular Ca^2+^ concentration increased from the leading edge to the rear in control scrambled cells in (Figures 7B,C). In contrast, this Ca^2+^ gradient was completely abolished in syndecan-4-knockdown cells (Figures 7B,C). Since it has been reported that Fura Red tend to accumulate in the mitochondria (Thomas et al., 2000), we explored whether the punctate structures can be observed in the Ca^2+^ indicator-loaded cell are mitochondria. Either control or syndecan-4-silenced cells exhibited distinct distribution for the Ca^2+^ indicators and the mitochondrial dye MitoTracker Deep Red (Supplementary Figure 10), demonstrating that neither Fluo-4 nor Fura Red accumulated in the mitochondria in our experiments. To exclude the possibility that alteration in the green and red fluorescence ratios is due to redistribution of organelles, in which one Ca^2+^ indicator accumulated more than the other, we performed an analysis, in which high intensity pixels (2.5-fold over mean cellular fluorescence) were omitted. Similar results were obtained this way to that shown in Figure 7 and Supplementary Figure 10, demonstrating that indeed the intracellular front-rear Ca^2+^ gradient was diminished by syndecan-4-silencing. In summary, our findings demonstrate the essential role of syndecan-4 in cell polarity.

## Discussion

Cell migration is an essential component of several physiological and pathological processes, including tissue regeneration. During regeneration of the skeletal muscle tissue, myoblasts (i.e., activated satellite cells, skeletal muscle stem cells) proliferate, differentiate, migrate and fuse to form tubular, multi-nuclear myotubes. Accordingly, during muscle development and regeneration, myoblasts must be capable of migration to promote the cell–cell interactions and myoblast fusion required for muscle fiber formation. Syndecans, a family of transmembrane proteoglycans, have been reported to play crucial roles in tissue regeneration (Chung et al., 2016). We demonstrated previously that syndecan-4 could influence myoblast proliferation, as syndecan-4 silencing reduced cell cycle progression from the G1 to the S phase and reduced the formation of mature myostatin, a negative regulator of muscle growth (Keller-Pinter et al., 2018). Syndecan-4 knockout mice also exhibited a decreased capacity for skin wound repair and angiogenesis (Echtermeyer et al., 2001), as well as inability to regenerate skeletal muscle following cardiotoxin-induced muscle necrosis (Cornelison et al., 2004). In summary, syndecan-4 appears to play an essential role in skeletal muscle development and regeneration, although the exact mechanism underlying this phenomenon remains unclear (Cornelison et al., 2004). Moreover, little is known about the specific role of syndecan-4 in mammalian myoblast migration.

Syndecan-4 was shown previously to affect migration in various cell types, including fibroblasts (Bass et al., 2007), endothelial cells (Chaudhuri et al., 2005), and hepatic stellate cells (Yin et al., 2017). This proteoglycan may also contribute to disease development by influencing the migration of tumor cells, such as lung adenocarcinoma (Toba-Ichihashi et al., 2016) and hepatoma (Charni et al., 2009); dendritic cells in the context of allergic rhinitis (Polte et al., 2015) and B-cells in the context of arthritis (Endo et al., 2015). A role for syndecan-4 has also been implicated in trophoblast migration and, consequently, the pathogenesis of preeclampsia (Jeyarajah et al., 2019). Importantly, Shin et al. (2013) reported that syndecan-4 overexpression increased the migration of turkey satellite cells and increased the activation of RhoA GTPase, and these motile phenomena required the cytoplasmic domain of syndecan-4. Other studies observed reduced motility following syndecan-4 knockdown in different cell types, consistent with our current observations, whereas high syndecan-4 level promoted migration (Toba-Ichihashi et al., 2016; Yin et al., 2017; Jeyarajah et al., 2019). Previous analyses of C2C12 mouse myoblast cells revealed that syndecan-4 was the most prominent heparan sulfate proteoglycan in these cells when compared with syndecan-1, syndecan-2, syndecan-3, glypican, or perlecan (Keller-Pinter et al., 2018), thus suggesting an important role for syndecan-4 in this cell type. However, the observed upregulation of syndecan-1, syndecan-2, and syndecan-3 mRNAs after syndecan-4 silencing (Keller-Pinter et al., 2018) suggests that other members of the syndecan family may compensate at least partially for the loss of syndecan-4. Given the importance of syndecan-4 in cell migration and cytoskeletal organization, we hypothesized that this proteoglycan would affect cellular polarity, centrosome positioning, and intracellular Ca^2+^ distribution during cell migration. We recently reported that syndecan-4 affects random migration and the directional persistence of migration in C2C12 cells during 18 h movement (Becsky et al., 2020). Here we show the effect of syndecan-4 silencing on Ca^2+^ distribution, centrosome positioning, and actin nanostructure after 8 h directional migration following wound scratching. Interestingly, the average speed values of the migrating C2C12 cells were similar in the case of both random (Becsky et al., 2020) and directional migration.

Cell polarization and the associated rearrangement of the actin cytoskeleton and cell–matrix relationships are key factors in cell migration. In addition to the integrins, syndecan-4 plays a pivotal role in the formation of focal adhesions. Specifically, syndecan-4 directly binds fibronectin to promote cell adhesions, thereby affecting cell migration, whereas the syndecan-4/PKCα/RhoA signaling axis promotes focal adhesion formation (Matthews et al., 2008; Yin et al., 2017). Furthermore, the downregulation of syndecan-4 was shown to suppress integrin-mediated cell adhesion by inhibiting FAK phosphorylation (Qin et al., 2017). Moreover, the cytoplasmic domain of syndecan-4 interacts directly with α-actinin (Greene et al., 2003), leading to associations with other adhesion molecules, such as vinculin and zyxin (Cavalheiro et al., 2017), as well as the actin cytoskeleton (Choi et al., 2008). In a recent study on endothelial cells, syndecan-4 knockdown was shown to induce the decoupling of vinculin from F-actin filaments (Cavalheiro et al., 2017). Interestingly, the interaction of PKCα and α-actinin with syndecan-4 was shown to be reciprocal (Chaudhuri et al., 2005). Moreover, syndecan-4 has been identified as a binding partner of dynamin II GTPase via its PH domain, and the resultant complex is a key regulator of focal adhesion and stress fiber formation in migrating cells (Yoo et al., 2005). Therefore, syndecan-4 serves as a central mediator in focal adhesion formation by bridging the interactions between integrins, fibronectin and intracellular molecules. Here we showed, that both the number and size of FAK stained focal adhesions were decreased in syndecan-4 knockdown cells during migration. Consequently, the loss of syndecan-4 would affect cell motility via multiple mechanisms, including the observed changes in the lamellipodial actin cytoskeletal structure.

As noted above, intracellular Ca^2+^ plays a crucial role in cell migration. Both Ca^2+^ influx from the extracellular space via different plasma membrane Ca^2+^ channels and Ca^2+^ release from intracellular stores (primarily the endoplasmic reticulum) contribute to the cytosolic Ca^2+^ concentration. In addition to contractility, changes in the intracellular Ca^2+^ affect the activities of calmodulin-dependent enzymes and actin-crosslinking proteins, thus playing a key role in the assembly of adhesions and junctions. Migrating cells establish a front-to-rear Ca^2+^ gradient, which increases toward the rear of the cell. Importantly, our findings suggest that syndecan-4 influences the development of this Ca^2+^ gradient, as demonstrated by its absence in syndecan-4 knockdown cells in association with decreased migration.

Syndecan-4 was shown earlier to influence Ca^2+^ concentrations in different cell types. In podocytes, syndecan-4 knockdown reduced the cell surface expression of the transient receptor potential cation channel subfamily C member (TRPC) 6 channel and consequently reduced the Ca^2+^ concentration (Liu et al., 2012). In contrast, another study of fibroblasts reported that the TRPC7 Ca^2+^ channel was more likely to be open in the absence of syndecan-4, resulting in an increased Ca^2+^ concentration (Gopal et al., 2015). However, a direct interaction has not been reported between syndecan-4 and TRPC7 (Afratis et al., 2017). Furthermore, the single knockdown of syndecan-4 in HaCaT keratinocytes did not affect the Ca^2+^ concentration, whereas the simultaneous silencing of both syndecan-1 and syndecan-4 decreased the cytosolic Ca^2+^ concentration in a TRPC4 channel-dependent manner (Gopal et al., 2015).

The development of Ca^2+^ gradient and the phosphorylation of FAK (Tyr397) are important for focal adhesion assembly and disassembly. Signaling via syndecan-4 is required for focal adhesion formation (Woods and Couchman, 2001), and syndecan-4 favors FAK phosphorylation (Wilcox-Adelman et al., 2002). The accumulation of phospho-FAK on the frontal side has been investigated and demonstrated in previous studies (Swaney et al., 2006; Carey et al., 2016; Gonzalez Malagon et al., 2018). The polarized distribution of syndecan-4 can affect both Ca^2+^ gradient and local phospho-FAK level. Furthermore, low amount of syndecan-4 in the rear of the migrating cells can contribute to focal adhesion disassembly.

As noted above, the localization of the centrosome is an indicator of polarization in a migrating cell (Etienne-Manneville and Hall, 2001; Zhang and Wang, 2017). To our knowledge, our study is the first to evaluate the effects of syndecan-4 on centrosome positioning, the Ca^2+^ gradient, and the consequent effects on cell polarity. In our previous report of the role of syndecan-4 in cytokinesis, we demonstrated the polarized distribution of the phospho-Ser179 syndecan-4, which accumulated in the intercellular bridges during cytokinesis (Keller-Pinter et al., 2010). The role for syndecan-4 in regulating the activity of RhoA and Rac1 had previously described (Bass et al., 2007; Keller-Pinter et al., 2017), which are crucial regulators of cell polarity. Here we demonstrated that syndecan-4 knockdown led to centrosome disorientation, which indicated improper cell polarization. Further studies are needed to determine the signaling processes leading to syndecan-4-dependent centrosome orientation. As the orientation of the centrosome-nucleus axis depends on a balance of actin- and microtubule-mediated forces (Elric and Etienne-Manneville, 2014), structural changes in the actin cytoskeleton may contribute to the observed mislocalization of centrosomes. Furthermore, changes in the quantity and, presumably, the localization of Rac1 GTPase in syndecan-4-knockdown cells may also affect centrosome positioning and polarity. The latter postulation is supported by an earlier observation that Rac1 activity and membrane protrusions are localized to the leading edges of migrating syndecan-4-sufficient cells, resulting in persistent migration, whereas syndecan-4-null cells migrate randomly (Bass et al., 2007).

The front-to-rear cell polarity required for migration depends on the activities of various members of the small GTPase Rho family. The rear of a migrating cell is defined by high levels of RhoA activity and subsequent actomyosin contractility, in addition to an increased Ca^2+^ concentration and the activation of Ca^2+^-dependent proteases required to cleave focal adhesion proteins. Interestingly, Tsai and colleagues suggested the presence of crosstalk between Ca^2+^ signaling and Rho GTPases that would coordinate the oscillations of these factors in the leading edges of migrating cells (Tsai et al., 2015). As noted, phospho-Ser179 syndecan-4 regulates both Rac1 GTPase activity (Keller-Pinter et al., 2017) and intracellular Ca^2+^ level (Gopal et al., 2015). It would be interesting to determine whether these processes are coordinated simultaneously by syndecan-4 during cell migration.

## Conclusion

**FIGURE 8:**
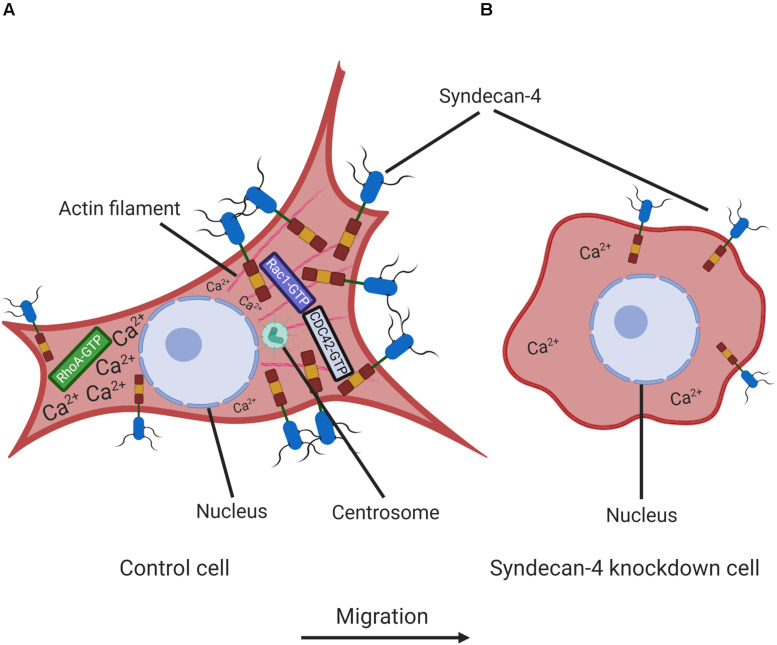
Schematic representation of the effect of syndecan-4 knockdown on cell polarity and migration. The intracellular distribution of Ca^2+^ and syndecan-4 in non-transfected control **(A)** and syndecan-4 knockdown **(B)** myoblasts. In migrating cells, the formation of protruding leading edges are driven by Rac1/CDC42-GTPases favoring the generation of focal contacts, while the retraction and detachment occur at rear edges driven by RhoA-GTP. Syndecan-4 distributes asymmetrically in migrating cells; and syndecan-4 knockdown resulted in the improper positioning of centrosomes, the absence of a front–rear Ca^2+^ gradient and disturbances in the nanoscale structures of the actin fibers. These abnormalities led to decreases in cell polarity and migration. The figure was created with BioRender.com.

In conclusion, we have identified new effects of syndecan-4 in the regulation of cell migration. Specifically, syndecan-4 silencing greatly reduces the migratory abilities of myoblasts. Presumably, this effect is due to a disturbance in cell polarization, which can be inferred from the shift in centrosome positioning relative to the nucleus and the absence of the intracellular Ca^2+^ gradient (Figure 8). The reduced migration capability might also be attributed to changes in the nanoscale structure of the lamellipodial actin cytoskeleton and reductions in cell–matrix adhesions. Our findings therefore elucidate the multiple roles of syndecan-4 in myoblast cell migration, although these findings are likely applicable to other cell types, given the ubiquitous expression of syndecan-4. This increase in general knowledge about cell migration will likely facilitate the development of strategies for the further exploration of a wide range of physiological and pathological migratory processes.

## Data Availability Statement

All datasets presented in this study are included in the article/Supplementary Material.

## Author Contributions

AK-P and LH conceived and designed the experiments. DB, KS, TG, SG-N, AB, ZB, LH, and AK-P performed the experiments. DB, KS, SG-N, LH, and AK-P analyzed the results. AK-P wrote the manuscript with inputs from DB, KS, SG-N, LH, TG, and ME. AK-P, ME, PH, LH, and LD edited the manuscript. DB and KS contributed equally to this work. AK-P was the principal investigator of the study. All authors contributed to the article and approved the submitted version.

## Conflict of Interest

The authors declare that the research was conducted in the absence of any commercial or financial relationships that could be construed as a potential conflict of interest.

## Footnotes

1 https://imagej.nih.gov/ij/

2 http://celltracker.website/
