# Supplementary material for: Syndecan-4 Modulates Cell Polarity and Migration by Influencing Centrosome Positioning and Intracellular Calcium Distribution
Source: Front Cell Dev Biol. 2020 Oct 15;8:575227. doi: 10.3389/fcell.2020.575227 (PMC7593626; doi:10.3389/fcell.2020.575227)
Supplement: Supplementary file 5 [file Data_Sheet_1.pdf]

SUPPLEMENTARY METHODS

Western blotting

Non-transfected, scrambled, and SDC4 knockdown (shSDC4#1 and shSDC4#2) cells were harvested in RIPA buffer (20 mM Tris-HCl pH 7.5, 150 mM NaCl, 1 mM Na2EDTA, 1 mM EGTA, 1% NP-40, 1% sodium deoxycholate, 2.5 mM sodium pyrophosphate, 1 mM glycerophosphate, 1 mM Na_3VO_4, 1 μg x ml leupeptin; Cell Signaling Technology, Danvers, MA, USA; #9806) supplemented with 1 mM Na-fluoride, and protease inhibitor cocktail (Sigma-Aldrich). The samples were spun down at 18,927 x g for 5 min at 4 °C to eliminate cellular debris, and the supernatants were separated by SDS/PAGE and blotted to nitrocellulose membrane. After blocking, membranes were incubated with primary antibodies including rabbit anti-SDC4 (PA1-32485; Pierce Protein Biology - Thermo Fisher Scientific, Waltham, Massachusetts, USA) and mouse anti-GAPDH (Cell Signaling Technology, #2118, Danvers, Massachusetts, US). Following incubation with the appropriate horse-radish peroxidase-conjugated anti-IgG secondary antibodies (DAKO, Glostrup, Denmark), peroxidase activity was visualized by enhanced chemi9luminescent method (Advansta, Menlo Park, CA, USA). Quantification of signal intensity was performed by QUANTITY ONE software (Bio-Rad, Hercules, CA, USA).

Immunostaining and quantification of focal adhesions

For the staining of focal adhesions, cells were fixed with 4% formaldehyde solution 2 and 4 h after scratching. After permeabilization with 0.3% Triton-X-100 (Sigma-Aldrich) and blocking in 3% BSA (Sigma-Aldrich), focal adhesions were stained with anti-FAK primary antibody (sc-271126; Santa Cruz Biotechnology, Dallas, TX, USA) and with an appropriate Alexa Fluor 488-conjugated secondary antibody (715-585-151; Jackson ImmunoResearch, Cambridgeshire, UK). Wide-field fluorescence images of FAK immunostained samples were acquired by a Nikon Eclipse Ti-E microscope (Nikon Instruments Inc.) with a 100× (Nikon CFI Plan Apo DM Lambda 100× Oil, NA = 1.45) objective, and processed using ImageJ software. The images were converted into binary images, particles in the range of 0 to 100 μm^2 were selected. The masks of these selected particles were constructed and the area of each particle was measured.

Assessment of mitochondrial accumulation of Ca^{2+} indicators

To explore the accumulation of Ca^{2+} indicators in the mitochondria, the cells were loaded by Ca^{2+} indicators as described in Materials and Methods, but in addition to the Ca^{2+} indicators, 100 nM MitoTracker Deep Red (Thermo Fisher Scientific) was included in the loading mixture.

The far red fluorescence of MitoTracker Deep Red was acquired above 650 nm at 633 nm excitation.
