# Supplementary material for: Syndecan-4 Modulates Cell Polarity and Migration by Influencing Centrosome Positioning and Intracellular Calcium Distribution
Source: Front Cell Dev Biol. 2020 Oct 15;8:575227. doi: 10.3389/fcell.2020.575227 (PMC7593626; doi:10.3389/fcell.2020.575227)
Supplement: Supplementary file 6 [file Data_Sheet_2.pdf]

SUPPLEMENTARY FIGURE LEGENDS

Supplementary Figure 1. Effect of syndecan-4 silencing on C2C12 myoblasts. (A) Representative Western blot analysis depicts the level of syndecan-4 in cells stably expressing shRNA against syndecan-4 (shSDC4#1, shSDC4#2), or scrambled shRNA. GAPDH served as a loading control. **(B)** Quantification of syndecan-4 expression in the different cell lines. n = 6 independent experiments, means + standard errors of the means; **: $p<0.01$; *: $p<0.05$.

Supplementary Figure 2. Distribution of focal adhesions in control and syndecan-4-silenced myoblasts. (A) Representative 100× wide-field fluorescence images depict the actin cytoskeleton (Alexa Fluor 647-conjugated phalloidin: red) and focal adhesion distribution (anti-FAK antibody: green) of the migrating cells in different cell lines 2 h after wounding. Nuclei are stained by Hoechst 33258 (blue). To quantify focal adhesions, the focal adhesions were assigned in FAK stained fluorescence images. The area and the number of the focal adhesions were quantified 2 and 4 h after wounding the monolayer **(B, C)**. Data are reported as means + standard errors of the means, n= 10 cells/cell line, 4 independent experiments were analyzed; *: $p < 0.05$; **: $p < 0.01$.

Supplementary Figure 3. Actin cytoskeletal structure of non-transfected C2C12 myoblasts around the cell-free zone. A representative wide-field fluorescence microscopy image depicts the actin skeletons of non-transfected C2C12 myoblasts around the cell-free zone. The inserts depict the lamellipodial actin structures in the migrating cells, which were imaged using dSTORM. The cells were fixed 2 h after confluent cultures were scratched, and the actin filaments were stained with Alexa Fluor 647-conjugated phalloidin (red). Nuclei are shown in blue (Hoechst 33258).

Supplementary Figure 4. Actin cytoskeletal structure of scrambled myoblasts around the cell-free zone. A representative wide-field fluorescence microscopy image depicts the actin skeletons of scrambled myoblasts around the cell-free zone. The inserts depict the lamellipodial actin structures in the migrating cells, which were imaged using dSTORM. The cells were fixed 2 h after confluent cultures were scratched, and the actin filaments were stained with Alexa Fluor 647-conjugated phalloidin (red). Nuclei are shown in blue (Hoechst 33258).

Supplementary Figure 5. Actin cytoskeletal structure of shSDC4#1 myoblasts around the cell-free zone. A representative wide-field fluorescence microscopy image depicts the actin skeletons of shSDC4#1 (i.e., syndecan-4 knockdown) myoblasts around the cell-free zone. The inserts depict the lamellipodial actin structures in the migrating cells, which were imaged using dSTORM. The cells were fixed 2 h after confluent cultures were scratched, and the actin filaments were stained with Alexa Fluor 647-conjugated phalloidin (red). Nuclei are shown in blue (Hoechst 33258).

Supplementary Figure 6. Actin cytoskeletal structure of shSDC4#2 myoblasts around the cell-free zone. A representative wide-field fluorescence microscopy image depicts the actin skeletons of shSDC4#2 (i.e., syndecan-4 knockdown) myoblasts around the cell-free zone. The inserts depict the lamellipodial actin structures in the migrating cells, which were imaged using dSTORM. The cells were fixed 2 h after confluent cultures were scratched, and the actin filaments were stained with Alexa Fluor 647-conjugated phalloidin (red). Nuclei are shown in blue (Hoechst 33258).

Supplementary Figure 7. Localization of centrosomes around the cell-free zone in the different cell lines 2 h after wounding. Representative wide-field fluorescence microscopy images depict the wounded areas in confluent cultures of non-transfected, scrambled and shSDC4#1 and shSDC4#2 (i.e. syndecan-4 knockdown) cell lines. The cells were fixed 2 h after the confluent cultures were scratched, and the centrosomes were stained with anti γ-tubulin (green). Nuclei are shown in blue (Hoechst 33258). Individual wide-field fluorescence images were obtained and used to generate panoramic maps for a further analysis of centrosome localization.

Supplementary Figure 8. Localization of centrosomes around the cell-free zone in the different cell lines 4 h after wounding. Representative wide-field fluorescence microscopy images depict the wounded areas in confluent cultures of non-transfected, scrambled and shSDC4#1 and shSDC4#2 (i.e. syndecan-4 knockdown) cell lines. The cells were fixed 4 h after the confluent cultures were scratched, and the centrosomes were stained with anti γ-tubulin (green). Nuclei are shown in blue (Hoechst 33258). Individual wide-field fluorescence images were obtained and used to generate panoramic maps for a further analysis of centrosome localization.

Supplementary Figure 9. Localization of centrosomes around the cell-free zone in the different cell lines 6 h after wounding. Representative wide-field fluorescence microscopy images depict the wounded areas in confluent cultures of non-transfected, scrambled and shSDC4#1 and shSDC4#2 (i.e. syndecan-4 knockdown) cell lines. The cells were fixed 6 h after the confluent cultures were scratched, and the centrosomes were stained with anti γ-tubulin (green). Nuclei are shown in blue (Hoechst 33258). Individual wide-field fluorescence images were obtained and used to generate panoramic maps for a further analysis of centrosome localization.

Supplementary Figure 10. Ca^{2+} indicators did not accumulate in the mitochondria. To explore the accumulation of Ca^{2+} indicators in the mitochondria, cells were loaded by Fura Red and Fluo-4 Ca^{2+} indicators and MitoTracker Deep Red to visualize mitochondria. **(A)** Representative confocal images depict the intracellular localization of Fura Red (red) and Fluo-4 (green), and MitoTracker Deep Red (blue) in scrambled, shSDC4#1, and shSDC4#2 cell lines. **(B)** Representative diagrams show the signal intensity of the different dyes along the migration axis in scrambled and syndecan-4 knockdown cells.
