# Supplementary material for: Syndecan-4 Modulates Cell Polarity and Migration by Influencing Centrosome Positioning and Intracellular Calcium Distribution
Source: Front Cell Dev Biol. 2020 Oct 15;8:575227. doi: 10.3389/fcell.2020.575227 (PMC7593626; doi:10.3389/fcell.2020.575227)
Supplement: Supplementary file 7 [file Data_Sheet_3.pdf]

SUPPLEMENTARY MOVIE LEGENDS

Supplementary Movie 1. The migration of C2C12 myoblasts into a cell-free zone. The migration of C2C12 cells was screened with live-cell microscopy. Images were acquired in 20 min intervals over an 8 h period after the removal of a cell culture insert. A representative greyscale time-lapse video is shown. The nuclei are stained with Hoechst 33342. One second in the movie is equivalent to 1 h of real time.

Supplementary Movie 2. The migration of scrambled myoblasts into a cell-free zone. The migration of scrambled C2C12 cells was screened with live-cell microscopy. Images were acquired in 20 min intervals over an 8 h period after the removal of a cell culture insert. A representative time-lapse video is shown. The nuclei are stained with Hoechst 33342. One second in the movie is equivalent to 1 h of real time.

Supplementary Movie 3. The migration of shSDC4#1 myoblasts into a cell-free zone. The migration of shSDC4#1 (i.e., syndecan-4 knockdown) cells was screened with live-cell microscopy. Images were acquired in 20 min intervals over an 8 h period after the removal of a cell culture insert. A representative time-lapse video is shown. The nuclei are stained with Hoechst 33342. One second in the movie is equivalent to 1 h of real time.

Supplementary Movie 4. The migration of shSDC4#2 myoblasts into a cell-free zone. The migration of shSDC4#2 (i.e., syndecan-4 knockdown) cells was screened with live-cell microscopy. Images were acquired in 20 min intervals over an 8 h period after the removal of a cell culture insert. A representative time-lapse video is shown. The nuclei are stained with Hoechst 33342. One second in the movie is equivalent to 1 h of real time.
