# Supplementary figures and images for: Syndecan-4 Modulates Cell Polarity and Migration by Influencing Centrosome Positioning and Intracellular Calcium Distribution
Source: Front Cell Dev Biol. 2020 Oct 15;8:575227. doi: 10.3389/fcell.2020.575227 (PMC7593626; doi:10.3389/fcell.2020.575227)

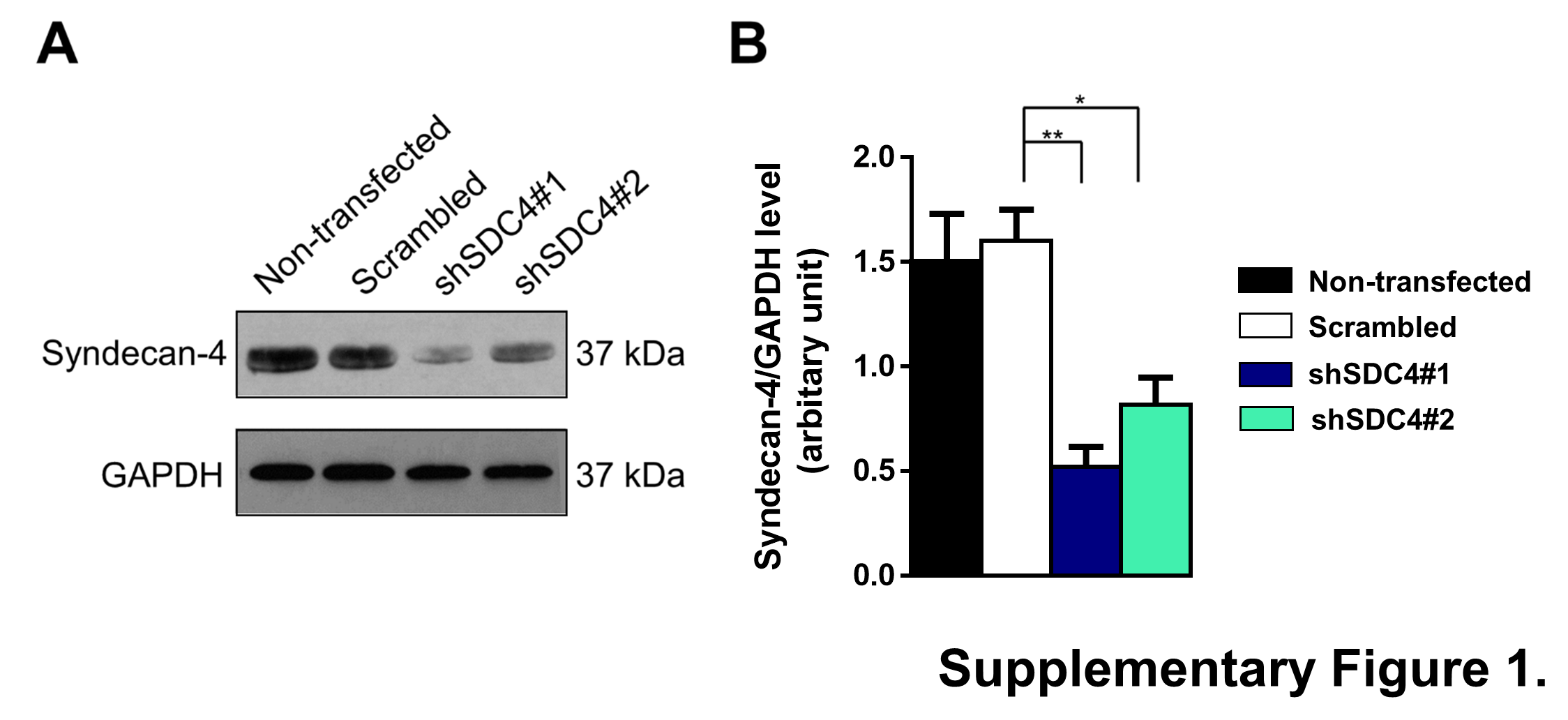

Supplement: Supplementary file 8 [file Image_1.TIF]

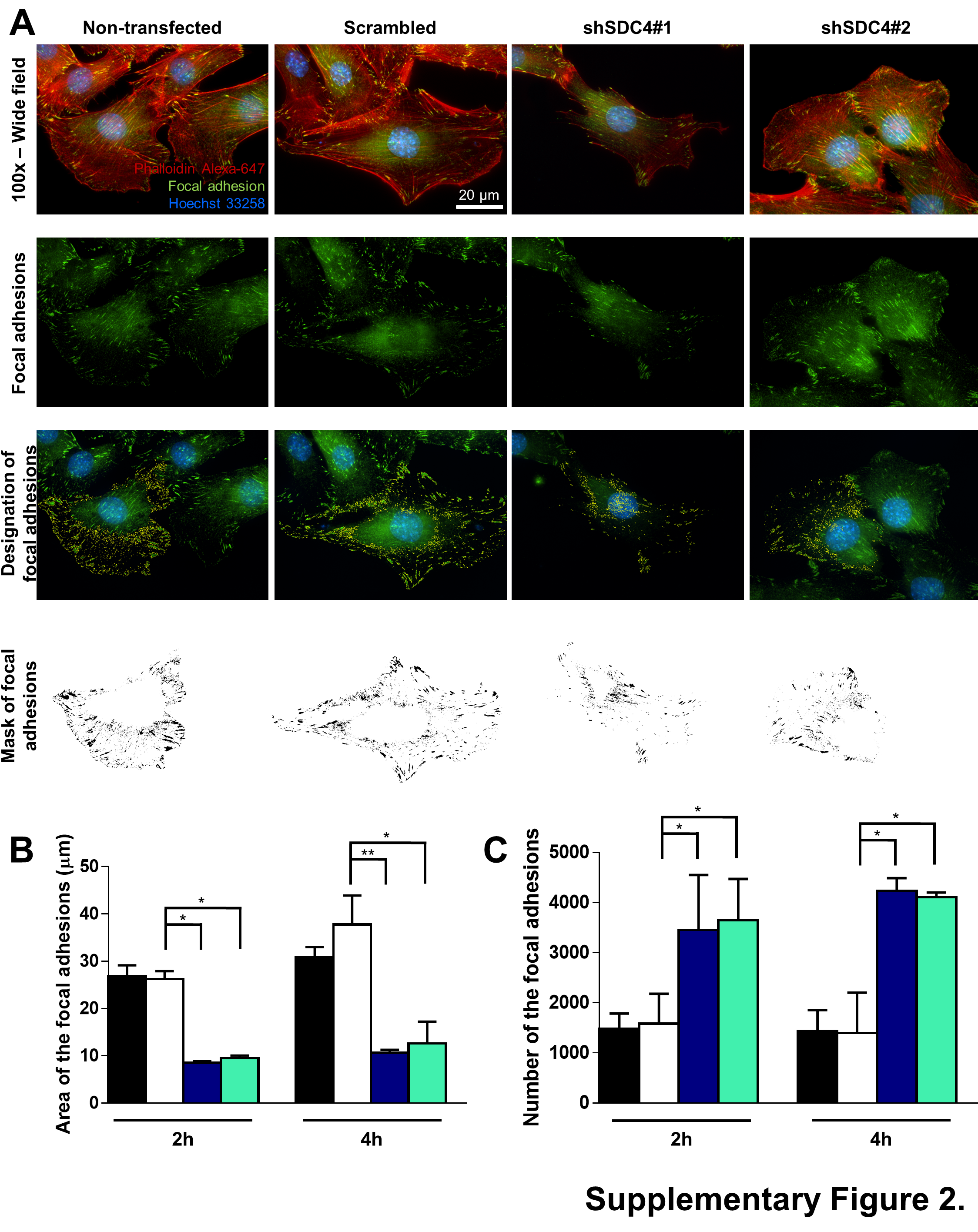

Supplement: Supplementary file 9 [file Image_2.TIF]

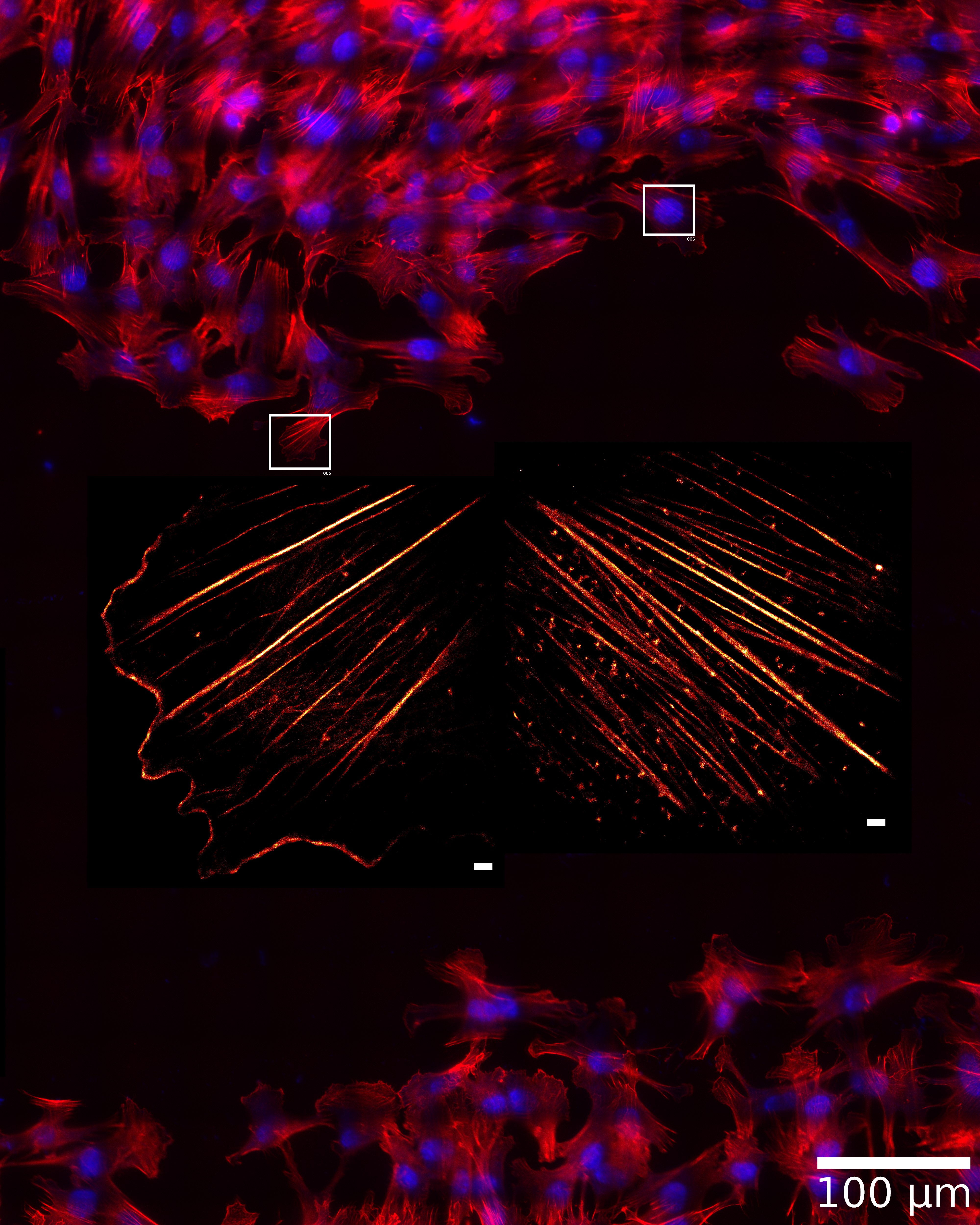

Supplement: Supplementary file 10 [file Image_3.png]

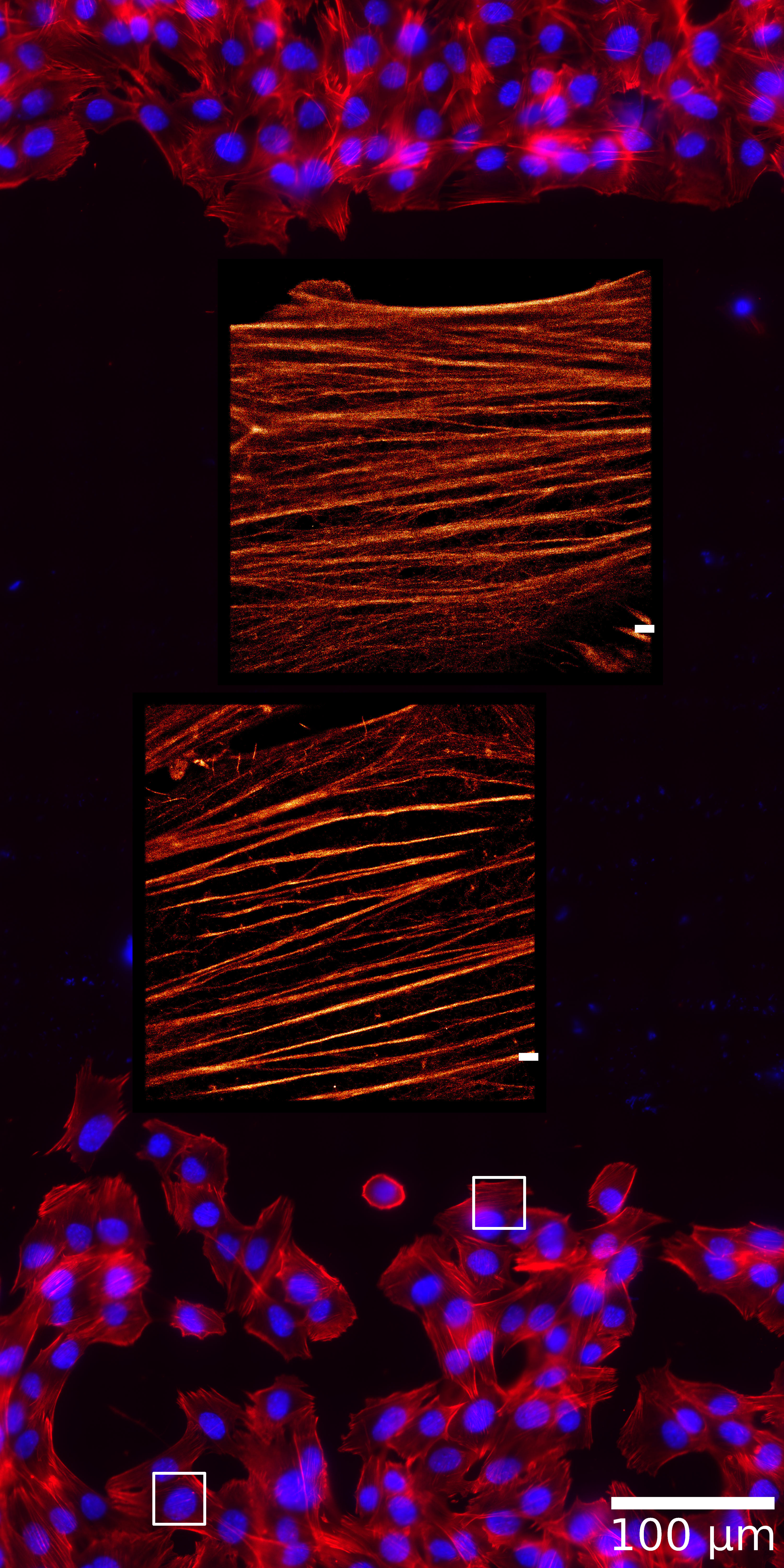

Supplement: Supplementary file 11 [file Image_4.png]

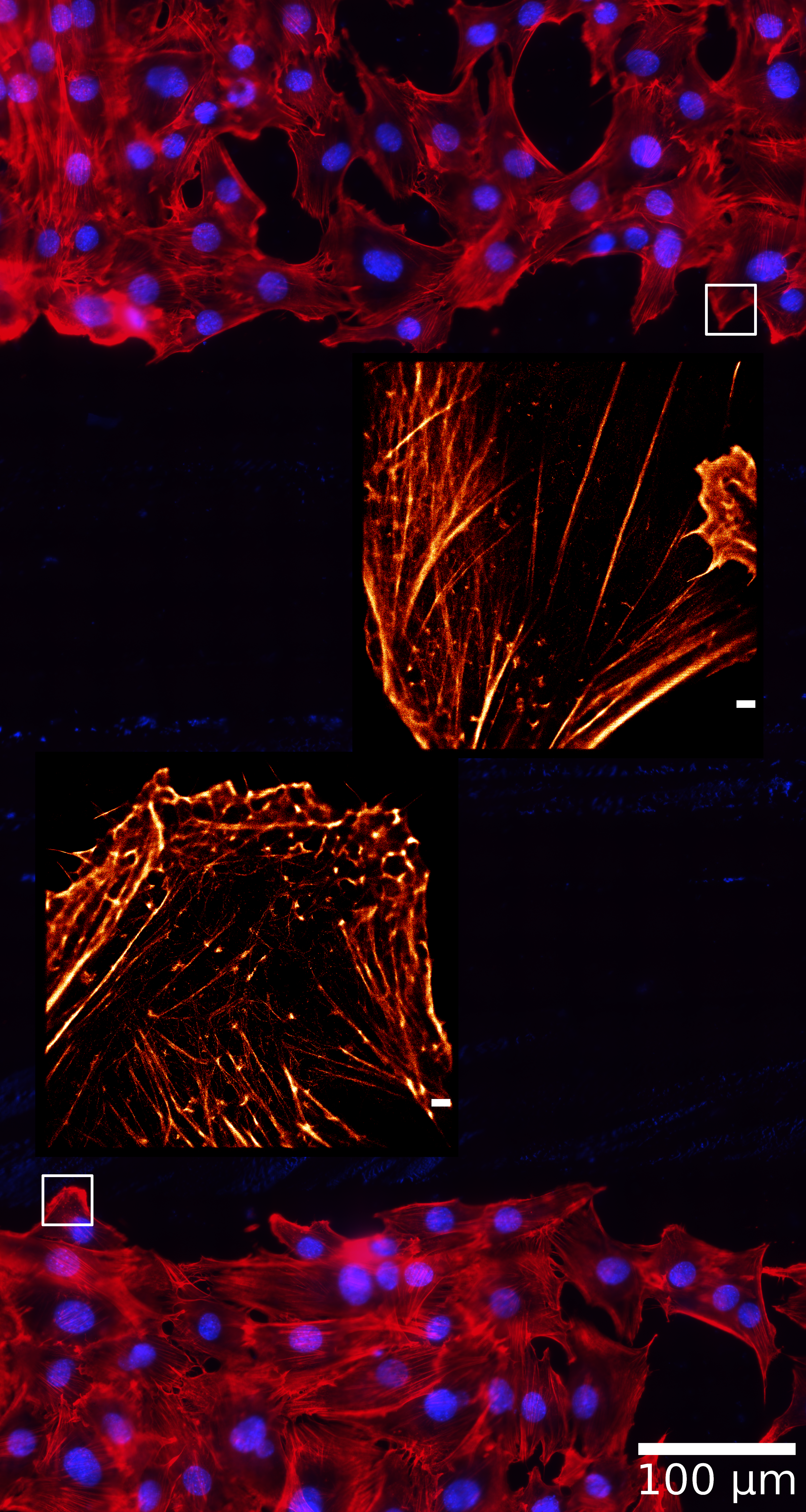

Supplement: Supplementary file 12 [file Image_5.png]

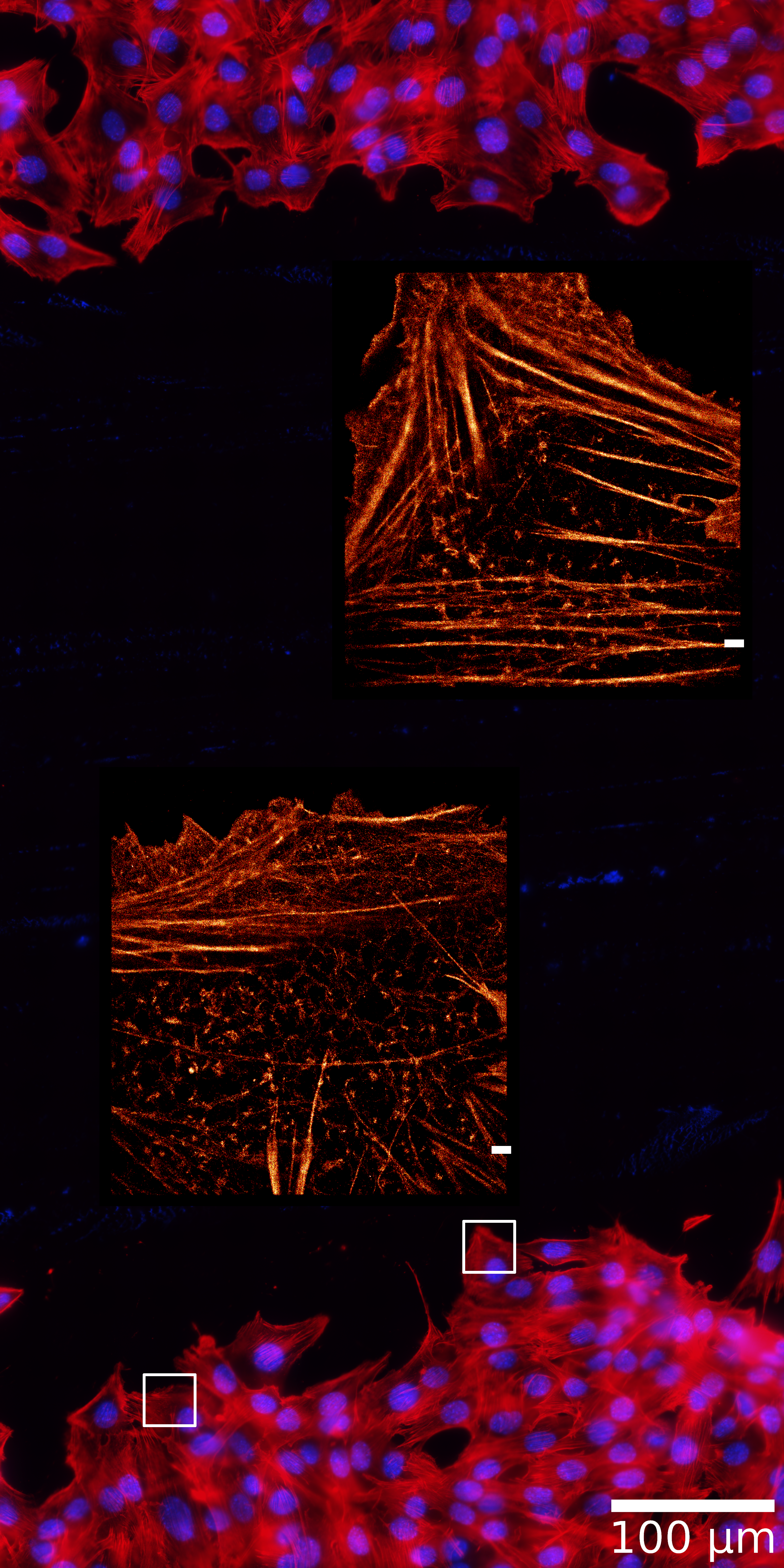

Supplement: Supplementary file 13 [file Image_6.png]

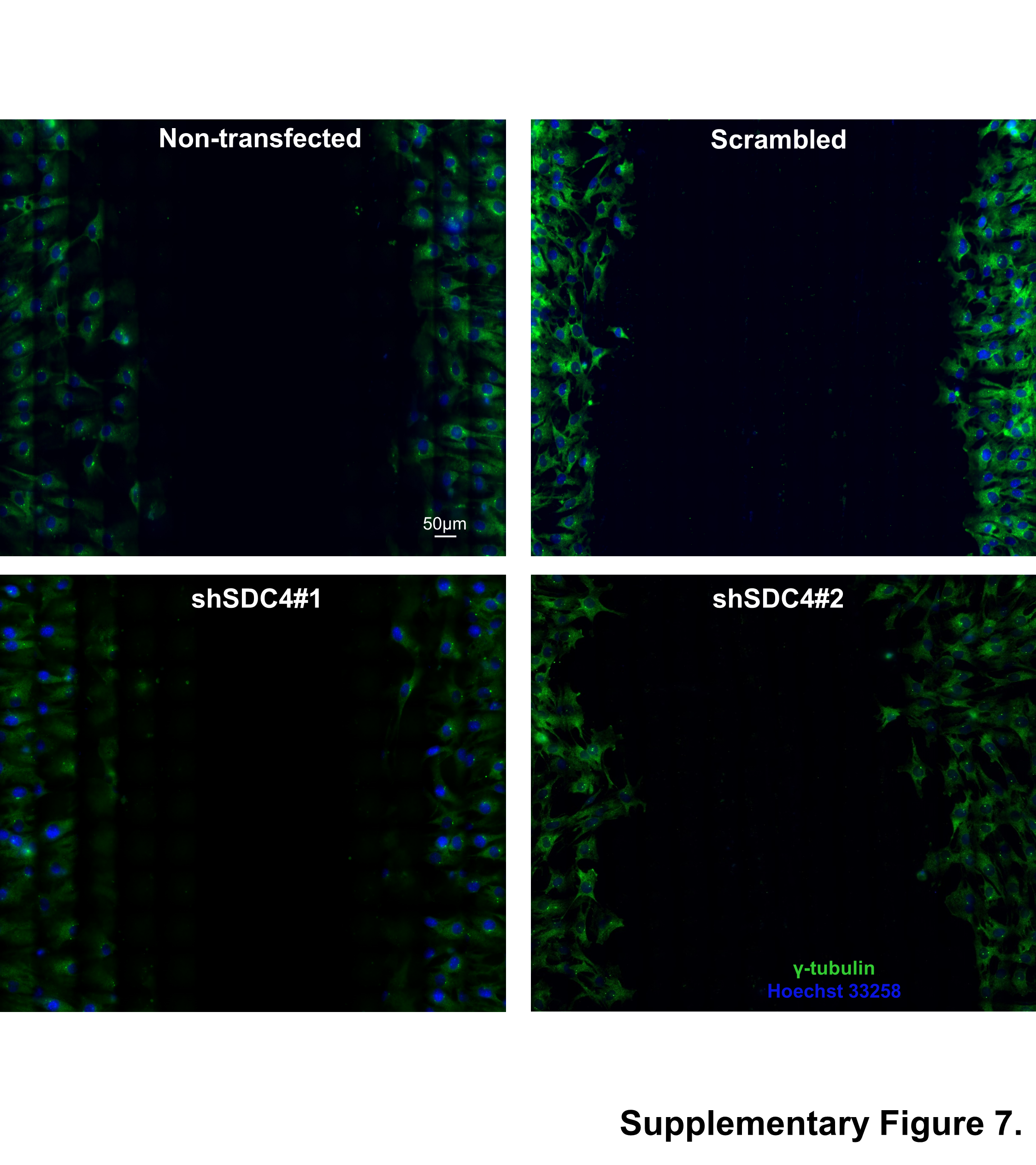

Supplement: Supplementary file 14 [file Image_7.TIF]

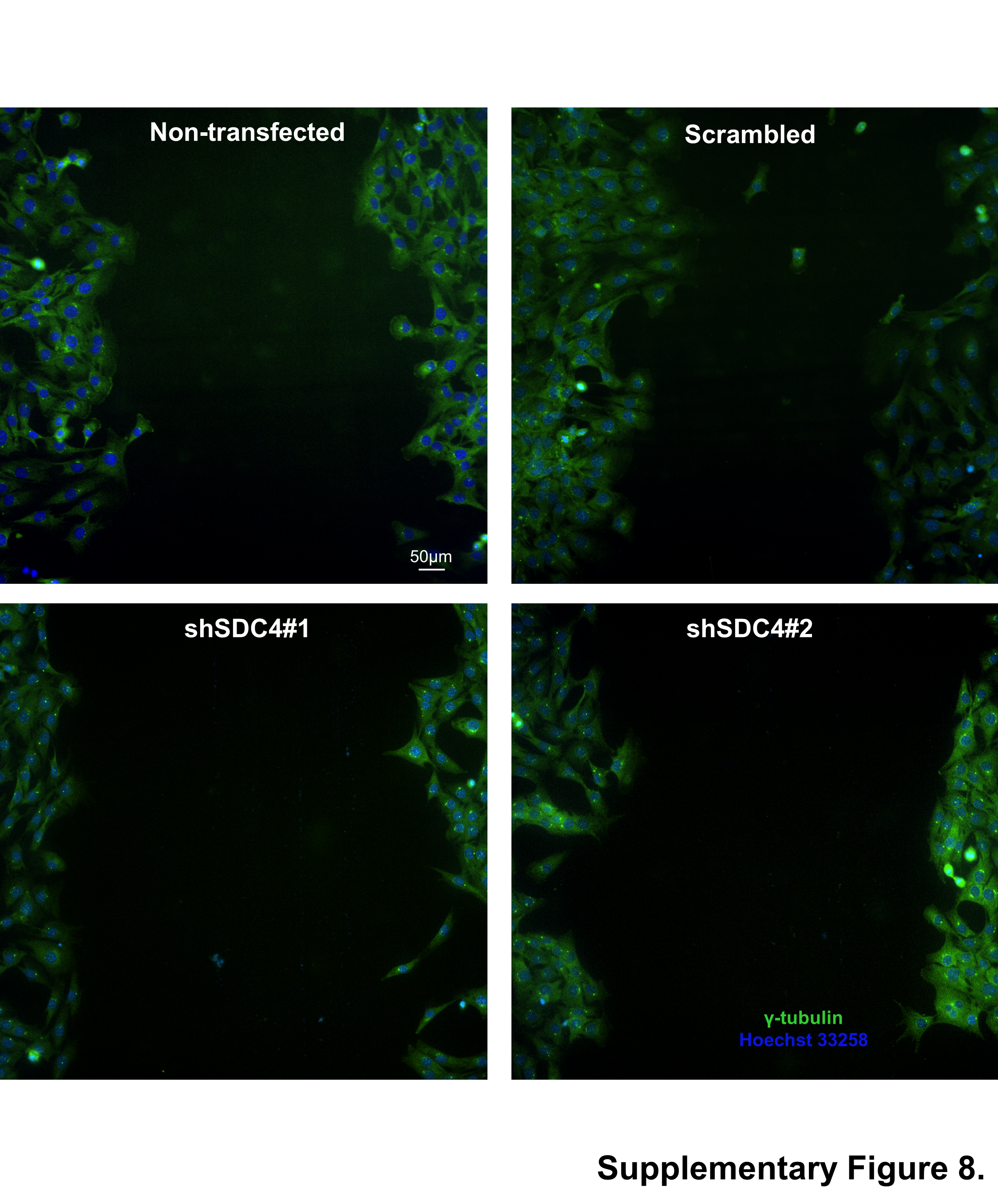

Supplement: Supplementary file 15 [file Image_8.TIF]

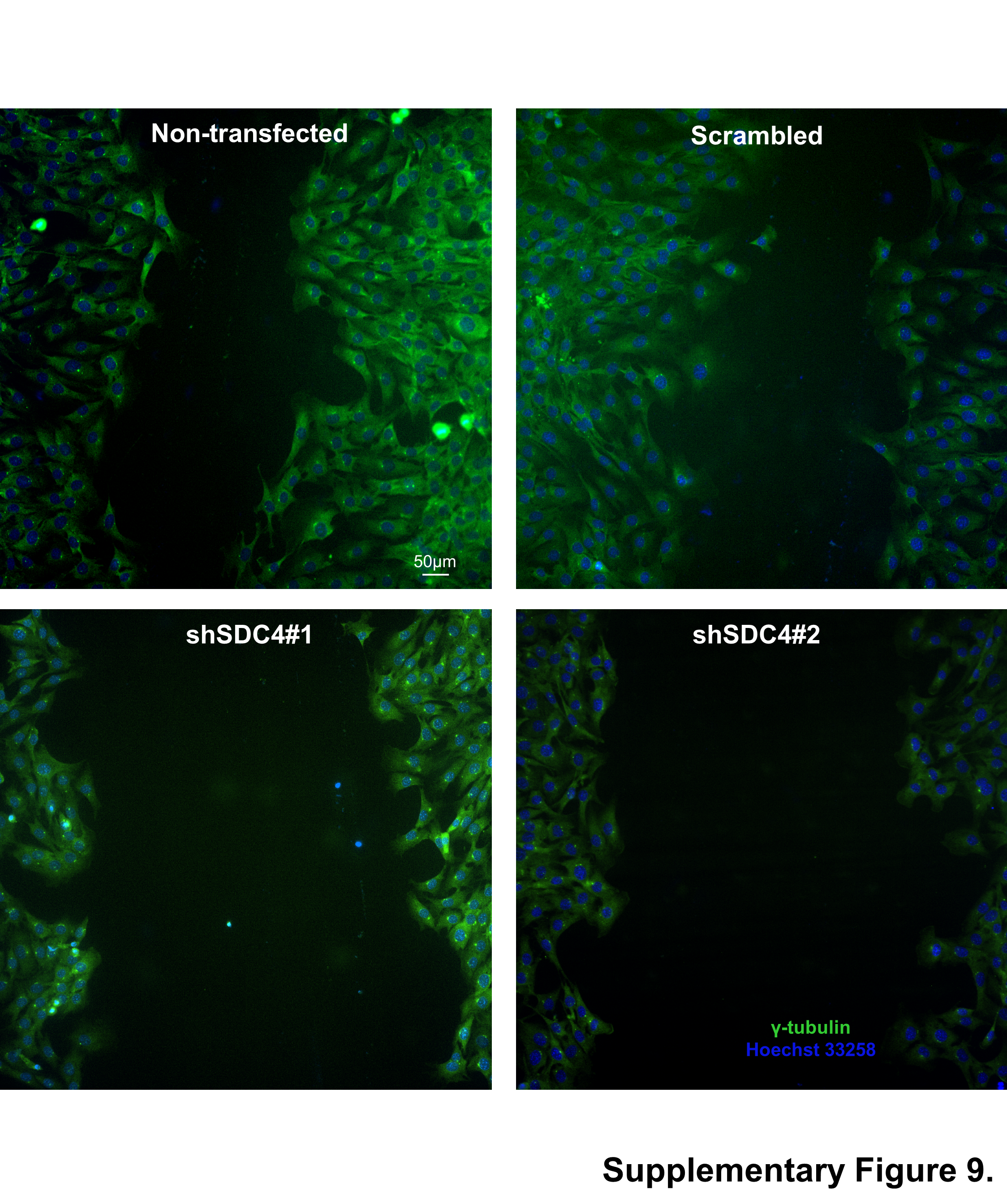

Supplement: Supplementary file 16 [file Image_9.TIF]

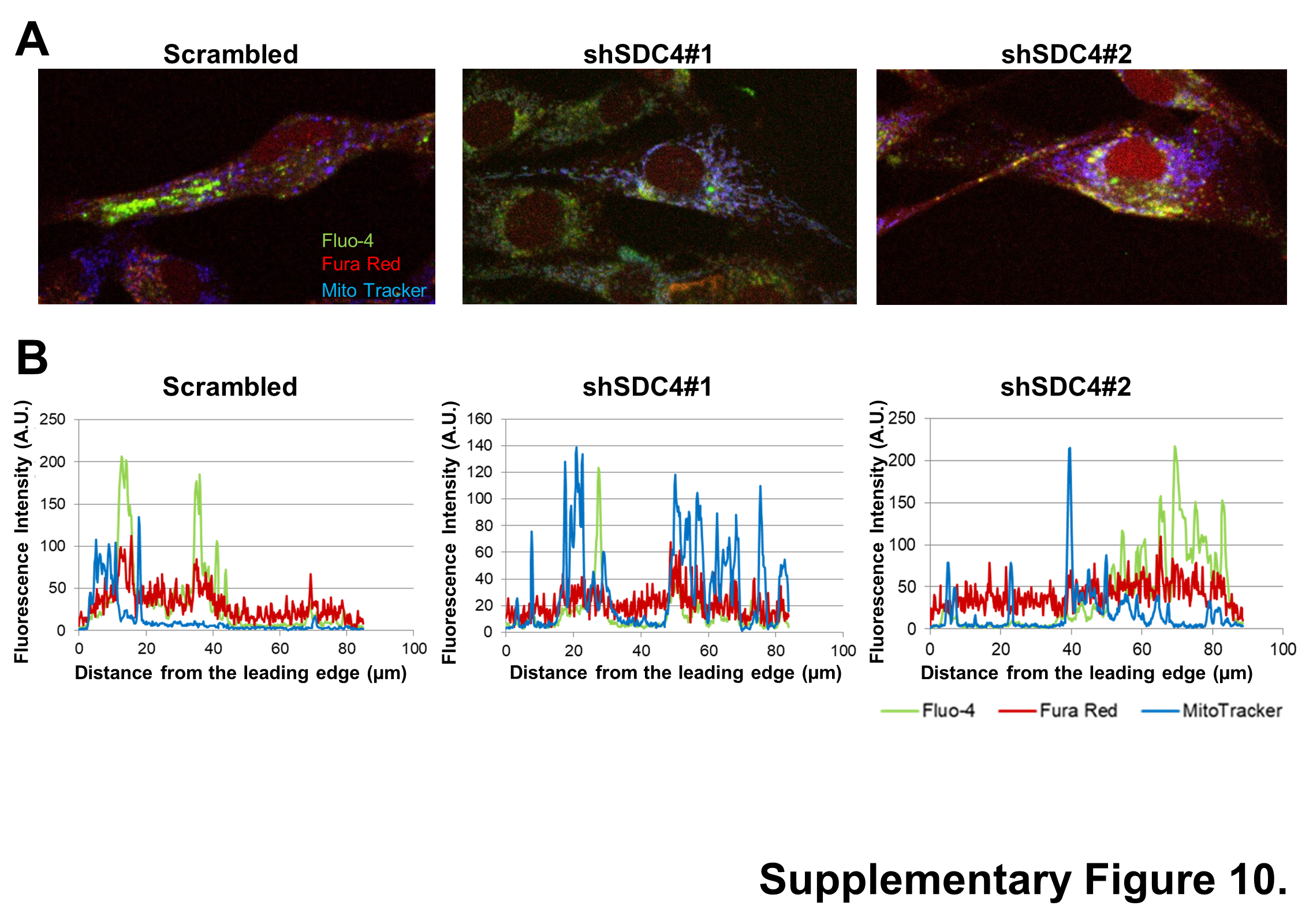

Supplement: Supplementary file 17 [file Image_10.TIF]
